# Improving reporting of meta-ethnography: the eMERGe reporting guidance

**DOI:** 10.1186/s12874-018-0600-0

**Published:** 2019-01-31

**Authors:** Emma F. France, Maggie Cunningham, Nicola Ring, Isabelle Uny, Edward A. S. Duncan, Ruth G. Jepson, Margaret Maxwell, Rachel J. Roberts, Ruth L. Turley, Andrew Booth, Nicky Britten, Kate Flemming, Ian Gallagher, Ruth Garside, Karin Hannes, Simon Lewin, George W. Noblit, Catherine Pope, James Thomas, Meredith Vanstone, Gina M. A. Higginbottom, Jane Noyes

**Affiliations:** 1 University of Stirling, Stirling, UK; 2 Edinburgh Napier University, Edinburgh, UK; 3 University of Edinburgh, Edinburgh, UK; 4 Cardiff University, Cardiff, UK; 5 Bangor University, Bangor, UK; 6 University of Sheffield, Sheffield, UK; 7 University of Exeter Medical School, Exeter, UK; 8 Department of Health Sciences, University of York, York, UK; 9 eMERGe project, Stirling, UK; 10 University of Leuven, Leuven, Belgium; 11 Global Health Unit Norwegian Institute of Public Health and Health Systems Research Unit, Oslo, Norway; 12 South African Medical Research Council, Capetown, South Africa; 13 University of North Carolina at Chapel Hill, Chapel Hill, USA; 14 University of Southampton, Southampton, UK; 15 EPPI-Centre Institute of Education, London, UK; 16 McMaster University, Hamilton, ON Canada; 17 NMAHP Research Unit, Unit 13 Scion House, Stirling University Innovation Park, Stirling, FK9 4NF UK; 18 School of Health Sciences & Centre for Evidence Based Health Care, The University of Nottingham, Nottingham, UK

**Keywords:** Guideline, Meta-ethnography, Nursing, Publication standards, Qualitative evidence synthesis, Qualitative research, Reporting, Research design, Systematic review

## Abstract

**Aims:**

The aim of this study was to provide guidance to improve the completeness and clarity of meta-ethnography reporting.

**Background:**

Evidence-based policy and practice require robust evidence syntheses which can further understanding of people’s experiences and associated social processes. Meta-ethnography is a rigorous seven-phase qualitative evidence synthesis methodology, developed by Noblit and Hare. Meta-ethnography is used widely in health research, but reporting is often poor quality and this discourages trust in and use of its findings. Meta-ethnography reporting guidance is needed to improve reporting quality.

**Design:**

The eMERGe study used a rigorous mixed-methods design and evidence-based methods to develop the novel reporting guidance and explanatory notes.

**Methods:**

The study, conducted from 2015 to 2017, comprised of: (1) a methodological systematic review of guidance for meta-ethnography conduct and reporting; (2) a review and audit of published meta-ethnographies to identify good practice principles; (3) international, multidisciplinary consensus-building processes to agree guidance content; (4) innovative development of the guidance and explanatory notes.

**Findings:**

Recommendations and good practice for all seven phases of meta-ethnography conduct and reporting were newly identified leading to 19 reporting criteria and accompanying detailed guidance.

**Conclusion:**

The bespoke eMERGe Reporting Guidance, which incorporates new methodological developments and advances the methodology, can help researchers to report the important aspects of meta-ethnography. Use of the guidance should raise reporting quality. Better reporting could make assessments of confidence in the findings more robust and increase use of meta-ethnography outputs to improve practice, policy, and service user outcomes in health and other fields. This is the first tailored reporting guideline for meta-ethnography. This article is being simultaneously published in the following journals: *Journal of Advanced Nursing, Psycho-oncology, Review of Education,* and *BMC Medical Research Methodology.*

**Electronic supplementary material:**

The online version of this article (10.1186/s12874-018-0600-0) contains supplementary material, which is available to authorized users.

## Why is this research or review needed?

- No bespoke reporting guidance exists for meta-ethnography, one of the most commonly used yet often poorly reported, methodologies for qualitative evidence synthesis which could contribute robust evidence for policy and practice.
- Existing generic guidance for reporting qualitative evidence syntheses pays insufficient attention to reporting the complex synthesis processes of meta-ethnography—tailored guidance should improve reporting and could improve quality of conduct.
- Better reporting of meta-ethnographies will likely have greater impact on understanding of specific phenomena of interest which will subsequently inform intervention development and changes in policy and practice.

## What are the key findings?

- Recommendations, guidance, and good practice for conducting and/or reporting all seven phases of a meta-ethnography were identified for the first time, along with uncertainties and evidence gaps regarding good practices.
- Nineteen reporting criteria were developed including detailed guidance on Phases 3–6: approach to reading/extracting data; processes for/ outcome of relating studies; processes for/ outcome of translation and synthesizing translations.
- The analysis and interpretation of methodological evidence and novel development work underpinning this new tailored reporting guidance advances meta-ethnography methodology, for example, to incorporate good practice in translation and synthesis.

## How should the findings be used to influence policy/practice/research/education?

- Use of the guidance by researchers, peer-reviewers, and journal editors to ensure complete and transparent reporting of meta-ethnographies will ensure their findings are optimized for use in policy and practice.
- The guidance can be used to inform the design and conduct of meta-ethnographies because of the underpinning rigorous, comprehensive analysis, interpretation, and synthesis of the latest methodological evidence.

## Introduction

Evidence-based decision-making for health services, policies, and programmes requires qualitative and quantitative research; this is recognized by leading evidence-producing organisations including Cochrane, the Campbell Collaboration, and the World Health Organization [1, 2]. To make sense of large volumes of research, robust syntheses of all types of research are needed [1]. Syntheses of qualitative studies, such as meta-ethnographies, can be used to develop theory about how a service, policy, strategy, or intervention works and how people experience these [3]; provide evidence of the acceptability, feasibility, and appropriateness of interventions or services [4–8]; convey people’s experiences of, for example, illness [9, 10]; and inform the development, implementation, and evaluation of complex interventions [11, 12].

### What is meta-ethnography?

Meta-ethnography is a seven phase, theory-based [13] and potentially theory-generating, interpretive methodology for qualitative evidence synthesis developed by sociologists Noblit and Hare [14] in the field of education. Meta-ethnography aims to produce novel interpretations that transcend individual study findings, rather than aggregate findings [15]. Meta-ethnography involves systematically comparing conceptual data from primary qualitative studies to identify and develop new overarching concepts, theories, and models. It was designed to preserve the original meanings and contexts of study concepts [9, 14].

The originators of meta-ethnography developed a distinctive analytic synthesis process of “translation” and “synthesis of translations” [14], underpinned by the theory of social comparison [13], which involves analysing the conceptual data, for example, concepts, themes, developed by authors of primary studies.

### Why is reporting guidance needed

Meta-ethnography is a distinct, complex and increasingly common and influential qualitative methodology. It is the most widely used qualitative evidence synthesis methodology in health and social care research [16–18] and is increasingly used by other academic disciplines [2]. Many other qualitative evidence synthesis methodologies and methods are based on or influenced by it [2, 19, 20]. A methodological evaluation of the effectiveness of meta-ethnography for synthesizing qualitative studies in health and health care concluded that meta-ethnography can lead to important new conceptual understandings of health care issues [9] and high quality meta-ethnographies have informed clinical guidelines [21, 22]. However, the quality of reporting in published meta-ethnographies varies and is often poor despite methodological advances [9, 17, 23–25]. Adequate quality in reporting is one of several prerequisites to assessing confidence in meta-ethnography findings that could inform evidence-based policy and practice, for instance, in health and social care [26].

Reporting guidance is commonly used in health and social care research and can raise publication standards [27]. For systematic reviews and meta-analyses of quantitative studies, the most commonly used guidance is Preferred Reporting Items for Systematic Reviews and Meta-Analyses (PRISMA) [28]. For reviews of qualitative studies, the most commonly used one is the generic 2012 ENTREQ (Enhancing transparency in reporting the synthesis of qualitative research) statement [29]. Qualitative evidence synthesis methodologies differ greatly; therefore, unique reporting guidance for metanarrative reviews was recently developed [30]. There is currently no guidance on reporting the complex synthesis process of meta-ethnography. Such guidance should improve the transparency and completeness of reporting and thus maximize the ability of meta-ethnographies to contribute robust evidence to health, social care, and other disciplines, such as education. Although meta-ethnography continues to evolve, reporting guidance is needed currently for this complex methodology.

## Methods

The methods used to develop the eMERGe meta-ethnography reporting guidance followed a rigorous approach consistent with, but exceeding, good practice recommendations [31] and were published in a protocol [32]. The research questions were:

1. What are the existing recommendations and guidance for conducting and reporting each process in a meta-ethnography and why? (Stage 1)
2. What good practice principles can we identify in meta-ethnography conduct and reporting to inform recommendations and guidance? (Stage 2)
3. From the good practice principles, what standards can we develop in meta-ethnography conduct and reporting to inform recommendations and guidance? (Stage 2)
4. What is the consensus of experts and other stakeholders on key standards and domains for reporting meta-ethnography in an abstract and main report/publication? (Stages 3 & 4).

**Fig 1:**
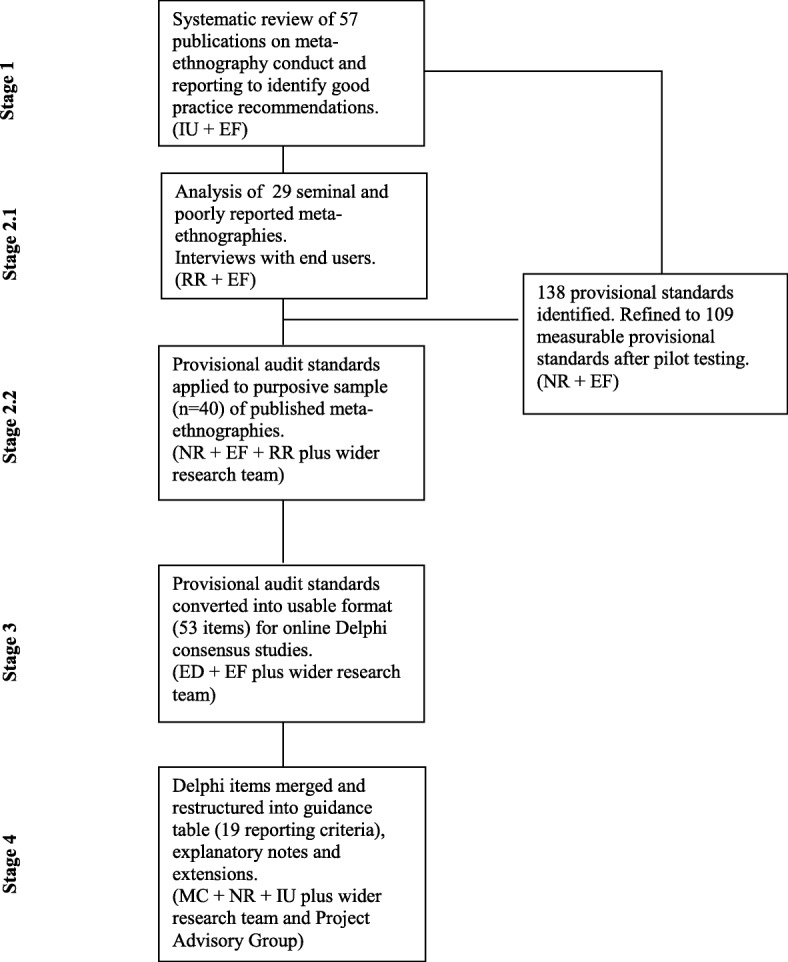
Guidance development flowchart

Details of the methods are given in supplementary File S1 (Additional file 1). Guidance development was conducted by the grant project team (the first 10 authors), in consultation with the one of the two originators of meta-ethnography, George Noblit and supported by a multidisciplinary project advisory group of national and international academics, policy experts, nonacademic users of syntheses such as clinical guideline developers and lay advisors, who had an active role in the development of the guidance and whose contributions were central throughout the project (the 11 authors from A. B. onwards were advisory group members). Guidance development took place over a 2-year period from 2015 to 2017 and comprised four stages, outlined in Fig 1:

1. Identification of potential reporting standards to include in the guidance;
2. Development and application of potential standards to published meta-ethnographies;
3. Consensus on guidance content;
4. Development of reporting criteria for the guidance and explanatory notes.

### Stage 1. Identification of standards

Stage 1 was conducted by the grant project team who undertook a systematic review (PROSPERO CRD42015024709) of relevant methodological and reporting guidance on meta-ethnographies to identify potential reporting standards [32]. From this review, we identified 138 recommendations for meta-ethnography standards on reporting from 57 included publications (see supplementary File S2) (Additional file 2).

### Stage 2. Development and application of the standards

The grant project team reviewed 29 published meta-ethnographies (see supplementary File S3) (Additional file 3) from various academic disciplines and interviewed nonacademic end users of meta-ethnographies to identify good practice principles and recommendations which we then developed into an audit tool of 109 measurable provisional standards. The 29 meta-ethnographies were chosen by academic experts who were asked to justify why they considered them seminal (i.e., they had influenced or significantly advanced thinking and/or were of central importance in the field of meta-ethnography) or relatively poorly reported, or meta-ethnographies were identified as poorly reported from published reviews. The team applied the provisional standards to a purposive sample of 40 published health and social care-related meta-ethnographies (selected from 571 identified through comprehensive systematic searches to give variation in, for example, journal, academic discipline, topic, number of included studies and of authors—supplementary File S1 gives full sampling details) (Additional file 1) in a retrospective audit to determine the extent to which the standards were met (“not at all”, “in part” or “in full”) and to identify ways the standards could be refined.

### Stage 3. Consensus on guidance content

From the results of Stage 2, the project team reviewed and refined the 109 provisional standards by clarifying ambiguous wording, merging duplicative standards, and combining standards on similar processes to create 53 items which were discussed in an online workshop and tested in Delphi consensus studies [33] with academic and nonacademic potential end users. Two parallel, online Delphi consensus studies with identical questions were conducted: one Delphi for international experts in qualitative methods (comprising editors or researchers with prior meta-ethnography/qualitative evidence synthesis experience) and one for professional/academic and lay people (potential end-users of meta-ethnographies). Sixty-two people (39 experts and 23 professional/lay people) completed all three rounds of the Delphi. Four items failed to reach consensus in both Delphi studies and so were excluded from the final guidance (these were the abstract should ideally differentiate between reported findings of the primary studies and of the synthesis; state the qualitative research expertise of reviewers; state in which order primary study accounts had data extracted from them; state the order in which studies were translated/synthesized). Participants reached consensus that 49 of 53 items should be included in the guidance, too many for usable reporting guidance; therefore, further steps were undertaken to condense these items into fewer reporting criteria.

### Stage 4. Development of the guidance

To develop the final reporting criteria for the guidance, a project advisory group meeting was convened which had 26 attendees including expert academics, other professionals, and lay members. The group discussed and agreed the structure of the guidance and the accompanying explanatory notes. Following this meeting, the grant project team agreed which Delphi items should be merged to create usable guidance. The project advisory group then commented on the readability and usability of the guidance. Members of the grant project team then further refined the guidance and explanatory notes. The final guidance and explanatory notes were checked against the Delphi items to ensure content and meaning had been preserved throughout this iterative process. Members of the project advisory group and project team reviewed and agreed the final guidance table and explanatory notes. Supplementary File S1 gives details of the methods which also appear in a published protocol [32] and funder’s report [34] (Additional file 1).

## How to use the guidance

The eMERGe reporting guidance is designed for use by researchers conducting a meta-ethnography (referred to throughout as “reviewers”: the term “reviewers” for people who conduct and report meta-ethnographies was the preferred term identified from the eMERGe Delphi studies in line with the increasing use of systematic review methodology for qualitative evidence syntheses), peer reviewers, journal editors, and end-users of meta-ethnographies including policy makers and practitioners. The eMERGe guidance also provides a helpful structure for anyone contemplating or conducting a meta-ethnography. While the guidance was developed for meta-ethnography, some of the reporting criteria, such as those relating to stating a review question and reporting literature search and selection strategies, might also be applicable to other forms of qualitative evidence synthesis and thus overlap with the generic ENTREQ guidance for reporting a wide range of qualitative evidence syntheses [29]. In contrast to eMERGe, ENTREQ does not provide guidance regarding reporting of the complex analytic synthesis processes (Phases 4–6) in a meta-ethnography and did not follow good practice guidance for developing a reporting guideline [31], for example, it was not designed with the consensus of a wider community of experts [34, 35].

The eMERGe guidance consists of three parts:

1. Part 1: Table of reporting criteria that are common to all meta-ethnographies,
2. Part 2: Detailed explanatory notes on how to apply the common reporting criteria including supplementary detail of findings for phases 3–6 (see supplementary information Table S4) (Additional file 4),
3. Part 3: Extensions for reporting steps and processes which are not common to every meta-ethnography.

Readers should refer to and use all three parts of the guidance. Parts 1 and 2 of the eMERGe reporting guidance are organized by the seven phases of meta-ethnography. Suggestions are provided in the grey cells of the table in Part 1 for where specific reporting criteria could be reported under journal article section headings. Where appropriate, reviewers should also consider additional relevant guidance for reporting other common qualitative evidence synthesis steps and processes, such as searches for evidence. See for example, the “STARLITE” guidance [36] and PRISMA [28] for reporting literature searches (refer to the EQUATOR Network for a comprehensive database of up-to-date reporting guidance https://www.equator-network.org/). Part 3 covers eMERGe extensions for format and content of the meta-ethnography output (for example, of an abstract); assessment of methodological strengths and limitations of included primary studies; and using the GRADE CERQual approach to assess confidence in findings from qualitative evidence syntheses [1, 26].

Users of this guidance should note that meta-ethnography is an iterative process and although the guidance is presented by meta-ethnography phases, we are not advocating a linear approach to meta-ethnography conduct. Furthermore, those conducting meta-ethnographies may need to be creative and adapt the methodology to their specific research/review question [37].

### Part 1: Guidance table (see Table 1)

**Table 1:** The eMERGe meta-ethnography reporting guidance

No.	Criteria Headings	Reporting Criteria
Phase 1—Selecting meta-ethnography and getting started		
*Introduction*		
1	Rationale and context for the meta-ethnography	Describe the gap in research or knowledge to be filled by the meta-ethnography, and the wider context of the meta-ethnography
2	Aim(s) of the meta-ethnography	Describe the meta-ethnography aim(s)
3	Focus of the meta-ethnography	Describe the meta-ethnography review question(s) (or objectives)
4	Rationale for using meta-ethnography	Explain why meta-ethnography was considered the most appropriate qualitative synthesis methodology
Phase 2—Deciding what is relevant		
*Methods*		
5	Search strategy	Describe the rationale for the literature search strategy
6	Search processes	Describe how the literature searching was carried out and by whom
7	Selecting primary studies	Describe the process of study screening and selection, and who was involved
*Findings*		
8	Outcome of study selection	Describe the results of study searches and screening
Phase 3—Reading included studies		
*Methods*		
9	Reading and data extraction approach	Describe the reading and data extraction method and processes
*Findings*		
10	Presenting characteristics of included studies	Describe characteristics of the included studies
Phase 4—Determining how studies are related		
*Methods*		
11	Process for determining how studies are related	Describe the methods and processes for determining how the included studies are related: - Which aspects of studies were compared AND - How the studies were compared
*Findings*		
12	Outcome of relating studies	Describe how studies relate to each other
Phase 5—Translating studies into one another		
*Methods*		
13	Process of translating studies	Describe the methods of translation**:** - Describe steps taken to preserve the context and meaning of the relationships between concepts within and across studies- Describe how the reciprocal and refutational translations were conducted- Describe how potential alternative interpretations or explanations were considered in the translations
*Findings*		
14	Outcome of translation	Describe the interpretive findings of the translation.
Phase 6—Synthesizing translations		
*Methods*		
15	Synthesis process	Describe the methods used to develop overarching concepts (“synthesised translations”)Describe how potential alternative interpretations or explanations were considered in the synthesis
*Findings*		
16	Outcome of synthesis process	Describe the new theory, conceptual framework, model, configuration, or interpretation of data developed from the synthesis
Phase 7—Expressing the synthesis		
*Discussion*		
17	Summary of findings	Summarize the main interpretive findings of the translation and synthesis and compare them to existing literature
18	Strengths, limitations, and reflexivity	Reflect on and describe the strengths and limitations of the synthesis: - Methodological aspects—for example, describe how the synthesis findings were influenced by the nature of the included studies and how the meta-ethnography was conducted.- Reflexivity—for example, the impact of the research team on the synthesis findings
19	Recommendations and conclusions	Describe the implications of the synthesis

### Part 2: Explanatory notes

#### PHASE 1—Selecting meta-ethnography and getting started

##### Reporting criterion 1—Rationale and context for the meta-ethnography

Consider whether a meta-ethnography of this topic is needed [38–40], for example, is there an existing meta-ethnography on the topic and if so, provide a reason for updating it [41] and describe the gap in research or knowledge to be filled by the meta-ethnography. This should include reviewers describing the availability of qualitative data which potentially could be synthesized and the context of the meta-ethnography, for instance, the political, cultural, social, policy, or other relevant contexts; any funding sources for the meta-ethnography; and the timescales for the meta-ethnography conduct. Reviewers should consider referring to frameworks which provide guidance on how to specify context, such as Noyes et al. [1].

##### Reporting criterion 2—Aim(s) of the meta-ethnography

The intention of meta-ethnography is to produce a new configuration/interpretation, a new model, conceptual framework, or theory, although ultimately this might not be possible, for instance, if no conceptual innovation had occurred since an early, conceptually rich primary study account [9, 42, 43]. The aim(s) of the meta-ethnography should be explicitly stated and should be compatible with such intentions. The aim may be refined after reading the literature and examining the available data [9, 24, 44–46]. If the initial aim(s) is (are) changed during Phases 1 and 2, give details of any refinements made.

##### Reporting criterion 3—Focus of the meta-ethnography

The review question(s) should be explicitly stated and be congruent with the intention of meta-ethnography. If, during later phases, the initial review question(s) or objective(s) needed to be refined, give details of any refinements. A well-defined review question, specifying a precise focus, can lead to a more efficient synthesis and more useful output [42, 45, 46], for instance, by contributing to clear study inclusion criteria for Phase 2.

##### Reporting criterion 4—Rationale for using meta-ethnography

Many qualitative evidence synthesis methodologies and methods exist [44]. Unlike meta-ethnography, some of these are aggregative (e.g., thematic analysis, Joanna Briggs Institute methods), combine qualitative *and* quantitative data (e.g., critical interpretive synthesis, metanarrative, metastudy, metasummary, realist synthesis), or have a realist epistemology (e.g., thematic synthesis, framework synthesis) [3, 20, 44]. The rationale should be given for why meta-ethnography was chosen as the most appropriate metet al.hodology for conducting an interpretive synthesis [40]. If reviewers made adaptations or modifications to Noblit and Hare’s [14] methodology or methods, state why meta-ethnography was still considered the most appropriate methodology and describe all adaptations and modifications made.

#### PHASE 2—Deciding what is relevant

##### Reporting criterion 5—Search strategy

Explain how the search strategy was informed by the research aim(s), question, or objectives and the meta-ethnography’s purpose [46, 47]. Reviewers should provide a rationale for whether the approach to searching was comprehensive (search strategies sought all available studies), purposeful (e.g., searching sought all available concepts until theoretical saturation was achieved), or a combination of approaches. Purposeful searches may be suited for theory-generating syntheses [46, 47]. In addition, provide a rationale for the selection of bibliographic databases and other sources of literature; when searching was stopped if purposeful searches were used; and any search limiters (restrictions to the searches) such as the years covered, geography, language, and so on.

##### Reporting criterion 6—Search processes

Describe and provide a rationale for how the literature searching was conducted, following appropriate guidance for reporting qualitative literature searches, for example, STARLITE [36], some journals may also require use of PRISMA [28].

##### Reporting criterion 7—Selecting primary studies

Describe the screening method, such as by title, abstract, and/or full text review and identify who was involved in study selection. Specify the inclusion and exclusion criteria for study selection, for example, in terms of population, language, year limits, type of publication, study type, methodology, epistemology, country, setting, type of qualitative data, methods, conceptual richness of data, and so on. Also, describe any sampling decisions for study selection—were all relevant studies included or a purposive or theoretical sample of studies [46, 48]?

##### Reporting criterion 8—Outcome of study selection

Provide details on the number of primary studies assessed for eligibility and included in the meta-ethnography. Give reasons for exclusion, for example, for comprehensive searches provide numbers of studies screened indicated in a figure/flowchart; for purposeful searching describe reasons for study exclusion and inclusion based on modifications to the review question and/or contribution to theory development.

Outcome of study selection can be presented as a primary study flow diagram or narrative—reviewers should note publication requirements—many journals require a PRISMA type flow diagram [28]. If comprehensive literature searches were conducted, reviewers should follow appropriate reporting guidance formats, such as PRISMA [28] and STARLITE [36]. If publication requirements prevent full reporting, reviewers should state where readers can access these data in full, for example, on a project website, in online files.

#### PHASE 3—Reading included studies

##### Reporting criterion 9—Reading and data extraction approach

This is the phase where the clearest divergence can start to be seen from other types of qualitative evidence syntheses. As described in the original meta-ethnography text:

“… we think it is best to identify this phase as the repeated reading of the accounts and the noting of interpretative metaphors. Meta-ethnography is the synthesis of texts; this requires extensive attention to the details in the accounts and what they tell you about your substantive concerns.” ([14], p. 28).

Reviewers should describe:

1. the process and strategy for reading included studies to indicate how close (critical) reading was achieved and who was involved in reading studies.
2. the strategy for extracting or recording data from included studies and state who was involved in this, whether processes were conducted independently by reviewers and whether data were checked for accuracy and if so, how.
3. the process for identifying and recording concepts, themes, and metaphors from the primary studies [25]. Indicate whether data were extracted from across the full primary study (desirable), or specific sections only, for example, findings (not recommended because conceptual data may appear throughout the account and the primary study context could be lost [37, 40]). Clarify which kind(s) of primary study findings were extracted, such as participant quotes and/or concepts developed by authors of primary studies (sometimes called first- and second-order constructs, respectively; [23]) so that readers can follow reviewers’ concept development.

Examples of how data extraction has been done include: create a list of metaphors and themes [9], create a grid or table of concepts [43, 49, 50], or code concepts in a software programme for the analysis of qualitative data such as QSR NVivo [40].

Reviewers should state what they mean by the terminology they have used for the units of synthesis, for example, metaphor, concept, theme.

##### Reporting criterion 10—Presenting characteristics of included studies

Provide a detailed description in narrative and/or table or other diagrammatic format of included studies and their study characteristics (such as year of publication, population, number of participants, data collection, methodology, analysis, research questions, study funder) [40, 49]. If publication requirements prevent full reporting, state where readers can access these data in full, for example, a project website, online files.

In addition, provide key contextual information about the primary studies and comment on their relevance to the context(s) specified in the meta-ethnography review question [42, 51, 52]. Context of included primary studies can influence the analysis process [42], for example, primary study accounts published after a certain date may reflect a change in health policy/practice such as the introduction of a smoking ban in enclosed public places. If two or more included primary study accounts, for example, papers, were derived from the same primary study, this should be made explicit. Contextual information should include details about the primary study participants (such as their gender, age, socioeconomic status, ethnicity, and so on); the setting such as a geographical setting (a country, region, city) or organisation (hospital, school, company, community); and key political, historical, and cultural factors of relevance, for instance, the introduction of a major international guideline, which affected clinical care, preceded publication of included studies. If such contextual information is not available in the primary study accounts, reviewers should make this clear to readers (Table 1).

#### PHASE 4—Determining how studies are related

##### Reporting criterion 11—Process for determining how studies are related

Reviewers should describe which aspects of the primary studies were compared and why, to determine how they are related, bearing in mind the aim of their meta-ethnography. Aspects could include: (i) research design, such as the: study aims; contexts; type of studies; theoretical approach/paradigm; participant characteristics, for example, their gender, ethnicity, culture, or age; study focus, for example, a health or social issue, long-term conditions, other diseases, or care settings; (ii) findings—the meaning of the concepts, metaphors, and/or themes [14]; the overarching storyline or explanation of a phenomenon from the primary study accounts [37] and (iii) other contextual factors, such as the time period, for instance, whether findings of primary study accounts differed because they were conducted in different time contexts. In addition, reviewers should describe how the studies were compared, that is, the methods and process of comparison. There is a wide variety of methods for comparing studies; examples of how Phase 4 has been reported include: Campbell et al. [24]; Atkins et al. [42]; Malpass et al. [43]; Beck [53]; Britten and Pope [49]; Erasmus [50].

##### Reporting criterion 12—Outcome of relating studies

Describe how primary studies relate: (i) to each other; (ii) to the review question; and (iii) to the prespecified aspects of context which were considered important, for example, do they relate reciprocally and/or refutationally, or do they explore different aspects of the topic under study [9, 14, 25, 42, 43, 49, 50, 53]? When reviewers are reporting how studies are related they should also report “disconfirming cases” [4, 51] that is, where one or more findings (e.g., metaphors or concepts) from a study differ from those of other studies for reasons that may be explained by differences in participants, settings, or study design. Reviewers can describe how studies were related in narrative, tabular, and/or diagrammatic form.

#### PHASE 5—Translating studies into one another

##### Reporting criterion 13—Process of translating studies

There is a variety of ways to conduct translation; therefore, reviewers should state their understanding and working definitions of reciprocal and refutational translation. Examples of approaches to translation identified by our systematic review are: Atkins et al. [42], Campbell et al. [9], Garside [54], Toye et al. [40], and Doyle [55]. Examples of refutational translation include Garside [54] and Wikberg and Bondas [56].

Reviewers should also:

1. state who was involved in translation;
2. describe how meaning was translated from one study into another, for instance, by reporting one or more examples of how this was done;
3. describe how relationships between concepts within and across studies, were preserved in the translation, such as by drawing concept maps to show relationships between concepts [43, 57] (grids, tables, and other visual diagrams could also be used);
4. describe how the contexts of the primary studies were preserved in the process of translation, for example, were subgroups of studies translated according to a common health condition or time-period [9]?
5. clearly indicate whose interpretation is being presented [25]—that of the research participants, study authors, or reviewers (sometimes called first-, second-, and third-order constructs, respectively) [23];
6. describe how potential alternative interpretations or explanations were considered in the translation.

Refutational translation is often overlooked [4, 51]; its purpose is to explain differences and to explore and explain exceptions, incongruities, and inconsistencies [47, 58]. An entire study could refute another study [49, 59] or concepts/metaphors within studies could refute one another [45, 49, 59], in which case it may be possible to do both reciprocal and refutational translation in a meta-ethnography rather than one or the other. Reviewers should identify disconfirming cases that could inform or have an impact on translation and, subsequently, synthesis.

Some argue that synthesizing a large number of studies might result in a superficial synthesis that loses its “groundedness” in the studies [9]; too few studies might result in underdeveloped theory/concepts [40, 45]. There is no consensus over what constitutes too few or too many studies; perceptions of a “large” number of studies varies from over 40 [9] to over 100 [51]. The volume of data will also depend on the richness and length of those accounts and team size will affect the ability to manage the data. If a large volume of data were synthesized, reviewers should explicitly describe how translation was achieved given this volume, for example, did they translate studies in smaller clusters to preserve conceptual richness and/or stay grounded in the data?

##### Reporting criterion 14—Outcome of translation

Describe the interpretive findings of the reciprocal translation and refutational translation—including how each primary study contributed to the translation [47] and describe alternative interpretations/explanations. Clearly document from which concepts in primary studies, the reviewers’ concepts are derived [47]. Reviewers need to differentiate between concepts derived from the participants of primary study accounts (sometimes called first order constructs) and those derived by the authors of the primary study accounts (sometimes called second-order constructs). An example of how this has been reported is Britten et al. [23] and a clear table describing the different levels of constructs can be found in Malpass et al. [43]. Descriptions of the study concepts and reviewers’ concepts and their interrelationships can be provided in table, diagrammatic or narrative form, with additional information in supplementary files. When quotes are used, reviewers should state their origin—primary study participants, primary study authors, or the reviewers’ own analysis notes. If any study was reported in more than one paper/account, describe how this was dealt with.

#### PHASE 6—Synthesizing translations

##### Reporting criterion 15—Synthesis process

There are two aspects of Phase 6: synthesizing translations and line of argument synthesis. The synthesized translations (concepts) represent the reviewers’ interpretation of the translations and are referred to in Britten et al. [23] as third-order constructs.

A line of argument synthesis aims to provide a fresh interpretation; it goes further than translation and puts any similarities and dissimilarities into a new interpretive context [14]. George Noblit [37] has more recently further defined a line of argument as the new “storyline” or overarching explanation of a phenomenon. Reviewers should describe the methods used to develop synthesized translations and how the line of argument synthesis was conducted. If line of argument synthesis was not conducted, state why not. In addition, describe:

1. how many and which studies were synthesized. Sometimes studies are excluded in Phases 5 and 6 (for instance, because they lack conceptual depth), so the number of synthesized studies may differ from the number of studies meeting review inclusion criteria.
2. who was involved in the synthesis and explain how synthesis findings have been considered from alternative perspectives (for example, from different academic disciplines) [42, 54, 59].
3. how reviewers remained grounded with primary study data and avoided losing conceptual richness during synthesis, particularly if a large amount of data were synthesized. (See the discussion on volume of data to be synthesized in Phase 5).

##### Reporting criterion 16—Outcome of synthesis process

Describe the interpretive findings of the synthesis of translations, the line of argument synthesis and any new model, conceptual framework or theory developed in a narrative, grid, table and/or visually, for instance, as an illustration, diagram or film. Any of these may be considered to be a synthesis product and a single synthesis may have more than one product. Reviewers should show the inter-relationships between the data from the primary studies and the reviewers’ new interpretations. If development of a new theory, conceptual framework, or model was not possible, state why not.

Describe the context where the new theory, model, or framework applies, or not, based on the characteristics of included primary studies. For example, the new theory may have been based solely on studies of young, white women, or studies conducted in countries with private health care, or the included studies may be older and/or predate a significant development in the field.

#### PHASE 7—Expressing the synthesis

##### Reporting criterion 17—Summary of findings

Relate the main interpretive findings to the synthesis objective(s), review question(s), focus, and intended audience(s) [9, 14, 42, 59, 60]. Compare the concept, model, or theory generated in the synthesis to the existing literature, such as research and policy publications. Reviewers should consider the possible influence of findings from other authors (both from primary study accounts and the wider literature) on their own conclusions [4].

##### Reporting criterion 18—Strengths, limitations, and reflexivity

Consideration of methodological and other strengths and limitations and how they may influence the final interpretation is a key to meta-ethnography reporting. Reviewers should reflect on and describe the effect of these on the synthesis process and outcomes because they may affect the credibility and trustworthiness (in other fields, this is referred to as validity and reliability) of the synthesis findings.

Strengths and limitations of: (i) the included primary studies; and (ii) how the meta-ethnography was conducted should be described. The latter are infrequently reported in published meta-ethnographies. Reviewers should comment on how these aspects may have influenced or limited the synthesis findings:

1. the characteristics, content and context of the primary studies, such as the temporal context, type of participant, cultural factors, study design.
2. the conduct of the synthesis. Considerations include, but are not restricted to: the order in which studies were synthesized [25, 54], the impact of study selection and sampling, the number of included studies/ volume of data (may affect depth of analysis), the context of the synthesis, and any modifications made to Noblit and Hare’s [14] original methodology.

Reflexivity—critically reflecting on the context of knowledge construction, especially the effect of the researcher on the research process—should include comment on how the reviewers influenced the interpretive process and synthesis findings [61], for example:

1. the reviewers’ background, perspectives, and experience, such as, but not limited to, epistemological position(s), professional position(s) held, academic discipline, organisation(s), or professional bodies represented [51];
2. if the reviewers have a specific view, stance, or personal interest, for example, the reviewer’s viewpoint on access to abortion care for a review about women’s reproductive health care services.
3. any influence of the funder of the meta-ethnography;
4. any conflicts of interests of the reviewers, that is, any factor, for example, financial, political, or organizational, which might influence the judgement of the reviewers when conducting the interpretation and synthesis.
5. how each reviewer was involved and how their contribution to literature searching and screening, reading of studies, data extraction, translation, and synthesis may have influenced the interpretive process [40, 42, 54, 59].

##### Reporting criterion 19—Recommendations and conclusions

Describe the implications of the synthesis findings for policy, practice, and/or theory. Policy and practice implicet al.ations were particularly important to eMERGe nonacademic and lay project advisors. Identify any areas where further primary or secondary research is needed.

### Part 3: Extensions

The first three extensions for reporting steps and processes that are not common to every meta-ethnography are available as supplementary material to this paper (see Additional file 5).

## Discussion

The eMERGe guidance is intended to increase transparency and completeness of reporting, making it easier for diverse stakeholders to judge the trustworthiness and credibility of meta-ethnographies and also intended to make the findings more usable and useful to inform services and interventions, such as in health, social care, and education. The development of this guidance used methods following, but exceeding, good practice in developing reporting guidance [31] incorporating systematic literature reviews; consensus methods; and consultation with one of the two originators of meta-ethnography, George Noblit. The team believe that the guidance is unusual among current reporting guidance in the extent to which it has involved lay people in all aspects of the development [32].

This guidance is not intended as a detailed guide in how to conduct a meta-ethnography—some such publications exist (e.g., [9, 41–43, 49]) and others from the eMERGe project are in preparation (see http://emergeproject.org/publications/). The guidance is designed to raise the reporting quality of meta-ethnographies and thus to assist those writing, reviewing, updating, and using meta-ethnographies in making judgements about quality of meta-ethnography conduct and output. It might also help users of qualitative evidence syntheses to recognize other forms of qualitative evidence synthesis mislabelled as a meta-ethnography, a common occurrence [25]. The guidance does, however, advance the methodology through its comprehensive analysis, interpretation and synthesis of methodological publications on meta-ethnography, published since Noblit and Hare’s original monograph, which underpin the reporting criteria and explanatory notes.

Some might argue that the guidance is overly prescriptive and detracts from the original purposes of meta-ethnography and, indeed, qualitative research. It is our view and that of others [62] who conducting a meta-ethnography involves creative, interpretive, qualitative analysis methods; however, a creative and interpretive approach should not preclude describing clearly how the research was conducted and some guidance is required to avoid misuse or mislabelling of the methods [15] and poor or misleading reporting. In this guidance, definitions and requirements have not been imposed arbitrarily, unnecessarily, or where consensus is lacking. Meta-ethnography has been described as an advanced qualitative research methodology [9, 38, 40] probably reflecting its complexity as a methodology. Training materials to accompany this guidance including video clips and slides (available from http://emergeproject.org/resources) have been developed as part of the eMERGe project.

This guidance has been designed to have the flexibility to be applied to diverse reporting formats with differing publication requirements (for example, journal articles, reports, book chapters) and this explains why some standards, which apply only to certain formats, are included as “extensions” to the guidance. Publication requirements can limit manuscript length; therefore, reviewers might need to provide some data in an alternative format, such as online, to achieve full reporting.

Methodological developments in meta-ethnography and in relevant qualitative evidence synthesis methodology generally will continue to occur. This guidance was created with an eye to accommodating these future developments which will be monitored through our discussion list: www.jiscmail.ac.uk/META-ETHNOGRAPHY. Future research will investigate the impact of the eMERGe reporting guidance, for example, by updating our earlier systematic review of meta-ethnography reporting practices [25], with a view to updating the guidance and we regard this guidance as one baseline from which to track the evolution of meta-ethnography.

## Conclusion

This guidance has been developed following a rigorous approach in line with and exceeding good practice in creating reporting guidance. It is intended to improve the clarity and completeness of reporting of meta-ethnographies to facilitate use of their findings to inform the design and delivery of services and interventions in health, social care, and other fields. Qualitative data are essential for conveying people’s (e.g., patients, carers, clinicians) experiences and understanding social processes and it is important that they contribute to the evidence base. Meta-ethnography is an evolving qualitative evidence synthesis methodology with huge potential to contribute evidence for policy and practice. In future, changes to the guidance might be required to encompass methodological advances and accommodate changes identified after evaluation of the impact of the guidance.

## Additional files

Additional file 1: **File S1.** Supplementary information: the eMERGe project research design & methods. (DOCX 184 kb)

Additional file 2: **File S2.** Supplementary information: A. 57 publications included in the systematic review for ‘Stage1 Identification of Standards’and B. Publications contributing to development of reporting criteria. (DOCX 299 kb)

Additional file 3: **File S3.** Supplementary information: the sample of 29 published meta-ethnographies analysed in ‘Stage 2 Development and Application of Standards’. (DOCX 27 kb)

Additional file 4: **Table S4.** Supplementary information: explanatory notes for Phases 3-6 to accompany Part 2 of the guidance. (DOCX 88 kb)

Additional file 5: Part 3: eMERGe Reporting Guidance - Extensions. (DOCX 48 kb)
