# Supplementary material for: Improving reporting of meta-ethnography: the eMERGe reporting guidance
Source: BMC Med Res Methodol. 2019 Jan 31;19:25. doi: 10.1186/s12874-018-0600-0 (PMC6359764; doi:10.1186/s12874-018-0600-0)
Supplement: Supplementary file 1 — File S1. Supplementary information: the eMERGe project research design & methods. (DOCX 184 kb) [file 12874_2018_600_MOESM1_ESM.docx]

File S1. Supplementary information: the eMERGe project research design & methods

Table of Contents

[Introduction 3](#_Toc517429058)

[Research questions 3](#_Toc517429059)

[Summary of design 3](#_Toc517429060)

[Stage 1 Methods 4](#_Toc517429061)

[Systematic review search strategy 4](#_Toc517429062)

[Comprehensive database searches and expansive searches 4](#_Toc517429063)

[Screening and selection of publications 5](#_Toc517429064)

[Data coding 6](#_Toc517429065)

[Data analysis 6](#_Toc517429066)

[Stage 2 Methods 6](#_Toc517429067)

[Stage 2.1 Documentary and interview analysis of seminal and poorly reported meta-ethnographies. 6](#_Toc517429068)

[Stage 2.1.a Analysis of seminal and poorly reported meta-ethnographies 6](#_Toc517429069)

[Stage 2.1.b Professional end-user views on utility of seminal and poorly reported meta-ethnographies for policy and practice 7](#_Toc517429070)

[Stage 2.2: Audit of published meta-ethnographies against provisional reporting standards. 8](#_Toc517429071)

[Audit methods 9](#_Toc517429072)

[Stage 3. Developing a consensus on the key standards for meta-ethnography reporting 12](#_Toc517429073)

[Aim 12](#_Toc517429074)

[Design 12](#_Toc517429075)

[Stage 3.1 Online expert and stakeholder workshop. 12](#_Toc517429076)

[Stage 3.2 eDelphi Consensus Studies 13](#_Toc517429077)

[Ethical approval 15](#_Toc517429078)

[Data collection 15](#_Toc517429079)

[Analysis 15](#_Toc517429080)

[Stage 4 Guidance Development Process 16](#_Toc517429081)

[References 17](#_Toc517429082)

[Appendix 1. PRISMA flow diagram for Stage 1 20](#_Toc517429083)

[Identification 20](file:///S:\a_NMAHP%20RU\eMERGe%20project\Draft%20&%20Final%20eMERGe%20Guidance%20Statement\The%20final%20guidance\eMERGe%20Guideline%20Revisions%2022June%202018\Supplementary%20information%20S1%2022June18.docx#_Toc517429084)

[Screening 20](file:///S:\a_NMAHP%20RU\eMERGe%20project\Draft%20&%20Final%20eMERGe%20Guidance%20Statement\The%20final%20guidance\eMERGe%20Guideline%20Revisions%2022June%202018\Supplementary%20information%20S1%2022June18.docx#_Toc517429085)

[Included 20](file:///S:\a_NMAHP%20RU\eMERGe%20project\Draft%20&%20Final%20eMERGe%20Guidance%20Statement\The%20final%20guidance\eMERGe%20Guideline%20Revisions%2022June%202018\Supplementary%20information%20S1%2022June18.docx#_Toc517429086)

[Eligibility 20](file:///S:\a_NMAHP%20RU\eMERGe%20project\Draft%20&%20Final%20eMERGe%20Guidance%20Statement\The%20final%20guidance\eMERGe%20Guideline%20Revisions%2022June%202018\Supplementary%20information%20S1%2022June18.docx#_Toc517429087)

[Appendix 2. PRISMA adapted flow diagram for Stage 2.2 21](#_Toc517429088)

[Screening 21](file:///S:\a_NMAHP%20RU\eMERGe%20project\Draft%20&%20Final%20eMERGe%20Guidance%20Statement\The%20final%20guidance\eMERGe%20Guideline%20Revisions%2022June%202018\Supplementary%20information%20S1%2022June18.docx#_Toc517429089)

[Sampling 21](file:///S:\a_NMAHP%20RU\eMERGe%20project\Draft%20&%20Final%20eMERGe%20Guidance%20Statement\The%20final%20guidance\eMERGe%20Guideline%20Revisions%2022June%202018\Supplementary%20information%20S1%2022June18.docx#_Toc517429090)

[Eligibility 21](file:///S:\a_NMAHP%20RU\eMERGe%20project\Draft%20&%20Final%20eMERGe%20Guidance%20Statement\The%20final%20guidance\eMERGe%20Guideline%20Revisions%2022June%202018\Supplementary%20information%20S1%2022June18.docx#_Toc517429091)

[Identification 21](file:///S:\a_NMAHP%20RU\eMERGe%20project\Draft%20&%20Final%20eMERGe%20Guidance%20Statement\The%20final%20guidance\eMERGe%20Guideline%20Revisions%2022June%202018\Supplementary%20information%20S1%2022June18.docx#_Toc517429092)

[Included 21](file:///S:\a_NMAHP%20RU\eMERGe%20project\Draft%20&%20Final%20eMERGe%20Guidance%20Statement\The%20final%20guidance\eMERGe%20Guideline%20Revisions%2022June%202018\Supplementary%20information%20S1%2022June18.docx#_Toc517429093)

# Introduction

This supplementary file contains a summary of the design and research methods used in the eMERGe project for developing the eMERGe reporting guidance; full details are published in a National Institute of Health (NIHR) project report.(1)

# Research questions

The eMERGe project research questions were:

1. What are the existing recommendations and guidance for conducting and reporting each process in a meta-ethnography, and why? (Stage 1)
2. What good practice principles can we identify in meta-ethnography conduct and reporting to inform recommendations and guidance? (Stage 2.1)
3. From the good practice principles, what standards can we develop in meta-ethnography conduct and reporting to inform recommendations and guidance? (Stage 2.2)
4. What is the consensus of experts and other stakeholders on key standards and domains for reporting meta-ethnography in an abstract and main report/publication? (Stage 3).(1)

# Summary of design

The project included four main stages (see Figure 1 in the main article), conducted by the project team, in consultation with one of the originators of meta-ethnography, George Noblit, and a Project Advisory Group of national and international academics, policy experts and lay people.(1) The design followed recommended good practice for creating reporting guidelines.(2)

Summary of stages 1-4:

- Stage 1 involved a systematic review of methodological guidance to identify good practice principles and recommendations.
- Stage 2 (2.1a) a documentary analysis of a sample of seminal and poorly reported published meta-ethnographies; (2.1b) interviews with professional end-users on the usefulness of those meta-ethnographies for policy and practice; (2.2) an audit of published health or social care related meta-ethnographies to identify if/how they met the good practice principles and recommendations identified in Stages 1 and 2.1 (a) and (b). We created 53 possible reporting items for the Delphi studies.
- Stage 3 involved seeking consensus on the reporting items through (3.1) an online workshop and (3.2) Delphi consensus studies.
- Stage 4 was to develop the guidance table, reporting criteria, explanatory notes, extensions to the guidance, and user training materials.

# Stage 1 Methods

A methodological systematic review (PROSPERO CRD42015024709) was conducted to identify guidance and recommendations for the conduct and reporting of meta-ethnography.

## Systematic review search strategy

Comprehensive database searches and ‘expansive’ searches were conducted. Relevant seminal methodological publications known to the eMERGe project team and its expert academic advisors were subject to citation searching and reference list checking. Details of databases and other sources which were searched are shown in Figure 1 and the search terms are shown in Table 1.

Figure 1. Databases and sources searched in Stage 1Methodological Review

| Databases   - MEDLINE (1947 to 2015) - Pubmed (inception to 2015) - International Bibliography of the Social Sciences (inception to 2015) - CINAHL (inception to 2015) - SCOPUS (1987 to 2015) - Web of Science Core Collection (inception to 2015) - PsycINFO (inception to 2015) - PsycARTICLES (inception to 2015) - Sociological abstracts (inception to 2015) - Applied Social Sciences Index and Abstracts (inception to 2015) - ERIC-Educational Resources Information Center) (inception to 2015) - British Education Index (inception to 2015) - Australian Education Index (inception to 2015)   Other sources   - **CRD (Centre for Reviews and Dissemination)** - Cochrane Collaboration - Open grey - Campbell Collaboration |
| --- |

## Comprehensive database searches and expansive searches

Sixteen bibliographic databases were searched in July and August 2015. Reference lists of publications included in the review were hand searched. Academic expert project advisors and team members also suggested publications. Endnote^®^ bibliographic software was used for reference management.

Table 1. Example of search terms used (for Scopus)

|  | **Scopus: >1987-Present Health Sciences/Social Sciences & Humanities TITLE-ABS-KEY** |
| --- | --- |
| 1 | ("qualitative synthes?s" or "qualitative systematic review*") |
| 2 | (meta-ethnograph* or metaethnograph* or meta-synth* or metasynth* or "line* of argument") |
| 3 | (("critical synth*" or "textual synth*" or "framework synth*" or "thematic synth*" or "grounded synth*" or "textual narrative synthes?s") W/2 (review*)) |
| 4 | (metasynthes?s or meta-synthes?s or meta-stud* or metastud*) |
| 5 | ((qualitative N/2 synth*) or ("third order" N/2 construct*) or (qualitative N/2 review*)) |
| 6 | “knowledge synthes?s” |
| 7 | or/1-6 |
| 8 | ((method* or steps) W/2 (insight* or lessons or learnt or explor* or learned or conduct* or approach*)) |
| 9 | “worked example*" |
| 10 | ((good or best or recommend* or quality or publishing or reporting) W/3 (guid* or design* or standard* or practi?e* or report* or method* or steps)) |
| 11 | “Lessons learnt” |
| 12 | ((challenges or steps) W/5 (synthesis* or qualitative or conduct* or report* or design* or method* or present* or practical*)) |
| 13 | (practical W/5 (guid* or design* or standard* or approach* or framework*)) |
| 14 | ((methods or methodological) W/5 (guid* or design* or standard* or approach* or framework*)) |
| 15 | **or/8-14** |
| 16 | **7 and 15** |

## Screening and selection of publications

9,332 references were identified from searches resulting in 7,522 after de-duplication. 6,271 (84%), published from 2006 to 2015, were independently double screened. One reviewer screened the remaining references, published before 2006, due to resource restraints. Expansive searches were used to identify any relevant publications published prior to 2006. Publications were screened by title, abstract and, when necessary, full text against the inclusion and exclusion criteria shown in Figure 2. A PRISMA diagram is given in Appendix 1.

Figure 2. Inclusion and exclusion criteria for Stage 1 systematic review

| Inclusion criteria | Exclusion criteria |
| --- | --- |
| - Book, book chapter, journal article/ editorial, report or PhD thesis - Published after 1988 - Reports on methodological issues* in conducting meta-ethnography OR Is a reporting guideline for or provides guidance on reporting qualitative syntheses including meta-ethnography - Any language - Any discipline or topic (not just health related) | - Theses below PhD level - Published before 1988 (date of the publication of the original meta-ethnography text by Noblit and Hare) - Does not report on methodological issues* in conducting meta-ethnography AND is not a reporting guideline/ providing guidance on reporting meta-ethnography |

*‘Methodological issues’ included all aspects of meta-ethnography methodology including: its philosophical and theoretical underpinnings; research design, practices and procedures including conveying findings and developing theory; providing advice on initial selection of meta-ethnography as suitable for one’s research aim, defining the characteristics of a meta-ethnography, comparing qualitative synthesis methodologies including meta-ethnography as one of those compared, and/or describing any other aspect of meta-ethnography methodology.(1)

## Data coding

Four reviewers, aided by a coding guidance document, coded advice and recommendations on how to conduct and report all aspects of a meta-ethnography from 57 full texts using NVivo 10.0 qualitative analysis software. One reviewer coded each publication; a second reviewer checked completeness of coding for 13 (23%) publications. Codes were mainly based on Noblit and Hare’s seven phases of meta-ethnography conduct.

## Data analysis

Coded data, with reference to the full publications when needed, were analysed qualitatively mainly by two reviewers using processes of constant comparison. Analysis for each node was recorded in analytic memos in NVivo. For complex phases or processes (e.g. Phases 4 to 6) each researcher independently identified key themes which were then compared. Each researcher kept an analysis journal and recorded whether the publications were “rich in detail” about meta-ethnography conduct and/or reporting, i.e. a detailed account with in-depth explanation and rationales that went beyond description. From the analysis, the researchers jointly wrote a detailed description of each phase of a meta-ethnography including advice, recommendations and documented pitfalls for their conduct and reporting, noting any contradictions or uncertainties. The initial findings were scrutinised and discussed by the wider team.

# Stage 2 Methods

## Stage 2.1 Documentary and interview analysis of seminal and poorly reported meta-ethnographies.

Stage 2.1 compromised of two stages: **(a)** documentary analysis of seminal and poorly reported meta-ethnographies, and **(b)** exploring professional end-user views on the utility of seminal and poorly reported meta-ethnographies for policy and practice.

### Stage 2.1.a Analysis of seminal and poorly reported meta-ethnographies

#### Methods

We intended to analyse 10–15 poorly reported and 10–15 seminal meta-ethnographies; in total we analysed 29 meta-ethnographies, 13 seminal and 16 poor. Expert academics from the eMERGe Project Advisory Group suggested meta-ethnography journal articles that they considered to be seminal (i.e. that have influenced or significantly advanced thinking and/or that are of central importance in the field of meta-ethnography) and those that they considered to be relatively poorly reported, and gave a rationale for their choices. The journal articles had to meet the following inclusion criteria:

- A peer-reviewed meta-ethnography journal article.
- Published following Noblit and Hare’s 1988 meta-ethnography book.
- Considered by our expert advisors and/or published reviews of meta-ethnographies to be either:
  - Seminal, or
  - relatively poorly reported.(3)

Only three poorly reported meta-ethnographies were suggested by experts, therefore, three published reviews(4-6) of meta-ethnography quality were searched by the project team identifying a further 13 poorly reported ones. In total, 13 seminal and 16 relatively poorly reported meta-ethnographies were analysed (see supplementary file S3 for a list of these).

##### Data Coding

Data were coded in NVivo 10.0(7) by three reviewers using a coding frame based on Noblit and Hare’s seven phases of meta-ethnography conduct, with additional codes for other important aspects of the methodology and its conduct, e.g. selecting a qualitative evidence synthesis approach, how to preserve the context of primary studies. The coded data were then compared to the recommendations identified in Stage 1.

##### Data Analysis

Focusing on phases 4 to 7, coded data for each phase were read repeatedly by one reviewer and systematically compared to the recommendations identified in Stage 1 to identify how they met/deviated from advice. The meta-ethnographies were also compared to one another. Preliminary findings were discussed regularly with the project team. This resulted in identification of similarities and differences between poorly reported and seminal meta-ethnographies.

### Stage 2.1.b Professional end-user views on utility of seminal and poorly reported meta-ethnographies for policy and practice

Meta-ethnographies can be used to inform policy and practice, therefore we included the views of potential end-users of meta-ethnographies (professionals not working in academia) on the usefulness of published meta-ethnographies to them in their professional role, to identify which aspects of reporting were important to them.

#### Methods

##### Sample

Individuals from relevant organisations were invited to participate if they met at least **one** of the following criteria:

- Works for a government or non-government organisation that uses synthesised evidence on health/social care, or develops or disseminates evidence-based health/social care guidance and advice
- Commissions qualitative evidence syntheses
- Works in a role related to the use of research evidence for health/social care policy or practice
- Clinical guideline developer
- Distils evidence for policy makers
- Health or social care policy maker
- Uses synthesised evidence or synthesises evidence in a professional non-academic capacity.(3)

##### Sample Recruitment

Twenty-three UK-based organisations were approached directly. In addition the Association of Medical Research Charities circulated an invitation to its 138 medical research charity members and the National Institute for Health Research (NIHR) circulated the invitation to its Board and Panel members. Eighteen organisations agreed to participate, of which 11 participated including non-departmental public bodies, medical research charities and Royal Colleges. Fourteen of their employees were interviewed, four more than our target. Only one participant had previously read a meta-ethnography.

##### Ethics

The interviews were exempt from research ethics approval.

##### Data Collection

Each participant was given one seminal and one poorly reported meta-ethnography, identified in Stage 2.1a, of relevance to them. Participants were not told which meta-ethnography was seminal or poorly reported. Semi-structured interviews were conducted with participants via telephone (n=13) or email (n=1) regarding the utility of the two meta-ethnographies. The interviewer took detailed notes during interviews.

##### Data Analysis

One team member conducted a thematic analysis of the interview data to identify professional end-users’ perceptions of good and poor reporting and the utility of meta-ethnography to inform policy and practice, as well as highlighting differences between the views of professional end-users and academics. Findings were discussed regularly by four project team members in analysis meetings, and with the wider project group at team meetings.

The combined findings of Stages 2.1a (documentary analysis of published meta-ethnographies) and 2.1b (interviews with potential end users of meta-ethnographies) enabled identification of good practice principles and contributed towards development of the reporting standards.

### Stage 2.2: Audit of published meta-ethnographies against provisional reporting standards.

Stage 2.2 involved (1) developing provisional reporting standards derived from the good practice principles and recommendations identified in Stages 1 and 2.1; and (2) auditing a sample of published health and/or social care-related meta-ethnographies against the provisional standards. The audit enabled refinement of the standards which contributed to the eventual reporting criteria.

#### Development **of provisional standards and audit tool**

The development of provisional standards was iterative. Every item of advice and recommended practice reported in Stage 1 and Stages 2.1 (a) and (b) was converted into a measurable draft standard. A bespoke audit tool was then created (see Table 2 below).

*Table 2. Excerpt from version 1 of the draft standards and audit reporting tool*

| **Advice/recommendations** | **Standard(s)** | **Evidence source(s)** |
| --- | --- | --- |
| *Phase 0 – Choosing meta-ethnography* | | |
| Many qualitative evidence synthesis approaches exist. Meta-ethnography should be considered and specifically chosen as the most appropriate interpretive methodological approach.  Meta-ethnography is suited to developing new conceptual understandings or new theories of experiences and/or behaviour especially when a topic is still being explored, developed and/or refined. | Meta-ethnography reports should have:  a clear rationale stating why meta-ethnography was considered the most appropriate qualitative evidence synthesis methodology | Stage 1 |
| \| **AUDIT TOOL (version 1)** \| \| \| \| \| \| \| \| --- \| --- \| --- \| --- \| --- \| --- \| --- \| \| **Standard number** \| *Phase 0 – Choosing meta-ethnography*  **Meta-ethnography reports should:** \| **Yes - in full** \| **Yes – in part** \| **No** \| **N/A** \| **comment** \| \| 0/1 \| report why meta-ethnography was considered the most appropriate qualitative evidence synthesis methodology \|  \|  \|  \|  \|  \| | | |

In refining the audit tool, duplicate standards were merged, ambiguous language clarified, the tool was piloted on published meta-ethnographies and revised resulting in a reduction from 138 to 109 provisional standards. The tool was formatted in in Microsoft^®^ Excel.^®^ Each standard could be recorded as fully met, partially met, not met or not applicable (N/A) with space for additional qualitative comments by auditors.

### Audit methods

Two team members led development of the provisional audit standards which were refined by all team members. Three members screened potential studies for inclusion in the audit. Six members audited sampled meta-ethnographies against the provisional standards in April 2016.

#### Identification of sample of meta-ethnographies for audit

A comprehensive systematic search for meta-ethnographies was carried out by one reviewer in six electronic databases (SCOPUS, Medline, EBSCO CINAHL, IBSS and Web of Science Core Collection) from their inception to 28 October 2015. Titles and abstracts were searched using the terms ‘meta ethnography’ or ‘metaethnography.’ A search for meta-ethnographies was conducted in the Cochrane register of qualitative evidence syntheses on 30 November 2015. The two sets of results were merged giving 1500 references which, after removing duplicates, resulted in 571 references - these were screened by title and abstract by one reviewer against the following inclusion/exclusion criteria:

Inclusion criteria

- Title, abstract and/or key words made reference to meta-ethnography or meta-ethnographic techniques or methods of Noblit and Hare.(8)
- Report of a synthesis of primary qualitative research studies.
- Had a health or social care-related focus.
- Published between 1994 and 2015 in English, French or Spanish.(3)

Exclusion criteria

- Title, abstract and/or key words made no reference to meta-ethnography or meta-ethnographic techniques or methods of Noblit and Hare.(8)
- Not a qualitative evidence synthesis, or, was a qualitative evidence synthesis but conducted using approaches other than meta-ethnography.
- Did not have a health or social care focus e.g. school education.
- Meta-ethnographies reported in languages that could not be translated by the team.
- Meta-ethnographies first-authored by members of the eMERGe Project Advisory Group and worked examples included in Stage 1 or Stage 2.1. (3)

Initial screening by title and abstract using the inclusion/exclusion criteria reduced the meta-ethnographies to a pool of 243 to which three team members applied further purposive sampling criteria so that the sample included meta-ethnographies:

- Published in a range of different journals e.g. medical, nursing, midwifery, allied health professional, social care or social science and at least one meta-ethnography in report rather than journal article format.
- Conducted by reviewers from different disciplinary backgrounds, different countries and from different philosophical traditions.
- Conducted by single and multiple reviewers.
- With a national or international primary studies e.g. included studies from different countries.
- That included different types of qualitative data.
- That were standalone or conducted alongside a quantitative systematic review.
- Represented a range in number of included studies e.g. less than 10, more than 50.
- Reviewers reported using ‘normal,’ ‘adapted’ or ‘modified’ meta-ethnography methods.(1)

The goal of purposive sampling was to ensure a diverse range of meta-ethnographies. The final selection of 40 eligible meta-ethnographies was made by the entire project team. However, when full texts were audited, 21 of these were not recognisable as a meta-ethnography, e.g. they combined qualitative and quantitative data or were literature reviews. These publications were excluded resulting in a final audit sample of 19 meta-ethnographies.(1) A PRISMA diagram is given in Appendix 2.

Table 3. Purposive sample of meta-ethnography publications audited

| Author(s) | Journal | Year |
| --- | --- | --- |
| Kane *et al.(9)* | Child Care Health & Development | 2007 |
| Ypinazar *et al. (10)* | Australian and New Zealand Journal Psychiatry | 2007 |
| Molony(11) | Research in Gerontology Nursing | 2010 |
| Purc-Stephenson *&* and Thrasher(12) | Journal of Advanced Nursing | 2010 |
| Wikberg and Bondas(13) | International Journal of Qualitative Studies Health and Wellbeing | 2010 |
| Malterud and Ulrikson(14) | International Journal of Qualitative Studies in Health Wellbeing | 2011 |
| Wells *et al.* ±(15) | (Research Report) | 2011 |
| Garrett *et al.(16)* | Chronic Illness | 2012 |
| Hoy(17) | International Journal of Men’s Health | 2012 |
| Monforte-Royo *et al.(18)* | PloS One | 2012 |
| Priddis *et al.(19)* | Journal of Advanced Nursing | 2013 |
| Sinnott *et al.(20)* | BMJ Open | 2013 |
| Soundy *et al.(21)* | Health Psychological Review | 2013 |
| Wells *et al.^±(22)^* | Psycho-Oncology | 2013 |
| Cullinan *et al. (23)* | Drugs and Aging | 2014 |
| Hole *et al.(24)* | Scientific world Journal | 2014 |
| Errasti-Ibarrondo *et al.(25)* | Nursing Outlook | 2015 |
| Galdas *et al.(26)* | Health Services Delivery & Research | 2015 |
| Lucas *et al.(27)* | Scandinavian Journal of Primary Health Care | 2015 |

#### Audit procedures

Each auditor was randomly assigned a selection of the meta-ethnographies. Verbal and written guidance was provided for use of the audit tool. A second auditor checked audit results with disagreements referred to a third auditor. For each standard, qualitative feedback from auditors was recorded.

#### Data analysis

One team member analysed audit data qualitatively and quantitatively . Descriptive statistics were prepared to identify how many provisional standards each publication met (in full, in part or not at all). All qualitative feedback was collated to identify standards which lacked clarity or were duplicative. Findings were discussed with the project team, for rigour and richer interpretation.

# Stage 3. Developing a consensus on the key standards for meta-ethnography reporting

## Aim

The aim of Stage 3 was to ascertain the consensus of meta-ethnography methodology experts and other key stakeholders on the key standards for reporting meta-ethnography in an abstract and main report or publication.

## Design

Stage 3 comprised two stages:

**Stage 3.1** Online expert and stakeholder workshop

**Stage 3.2** eDelphi Consensus Studies.

### Stage 3.1 Online expert and stakeholder workshop.

The workshop was essential for the reporting guidance development because it ensured that participants had the latest knowledge about meta-ethnography and the quality of its reporting. The workshop exceeded good practice in developing a reporting guideline(2) by including not just academic experts but a wide range of stakeholders including lay people.

#### Recruitment

Seventy-eight people were recruited to the workshop, 31 of whom participated: 12 academics, 3 other professional stakeholders, 11 lay people, and 5 project team members. A further nine project participants (six academics and three lay people) gave feedback on the workshop outputs after the workshop.(1)

#### Procedure

A three-hour online workshop took place on 12 May 2016. The project team and participants discussed good and best practice in meta-ethnography conduct and reporting, and further developed the draft reporting standards and their wording.

#### Process

An online conferencing system, Blackboard Collaborate™, was used to conduct the workshop. Presenting project team members had video enabled. Detailed workshop documents containing the main project findings to date, examples of the standards, a glossary of technical terms and an attendees list were circulated in advance. Summaries of the findings and standards were presented during the workshop.

#### Data collection and analysis

Following 25 minutes of presentations by two team members there was open discussion with all participants including discussing a range of draft standards. We explored the definition of a meta-ethnography, how close the draft standards were to best practice, and the utility of meta-ethnography reports for improving clinical practice and intervention implementation. Participants could suggest additional standards for inclusion in the eDelphi studies and suggest revisions to the draft standards. The workshop was audio-recorded and detailed notes, structured by discussion topic, were produced which were circulated for comment and amendments to all participants and to those who could not attend the workshop.

The reporting standards were revised as a result of the workshop but none was deleted because it was not the purpose of the workshop, but of the eDelphi, to select standards for the guidance. Finally, we presented our revised standards to George Noblit and discussed these with him in June 2016. This resulted in further refinements to the standards to clarify and improve their utility. The final list comprised **69** eDelphi items (**53** of which related to the content of a meta-ethnography publication, **16** related to potential journal headings and subheadings under which the content could be structured).

### Stage 3.2 eDelphi Consensus Studies

#### Objectives

The objective was to conduct two identical eDelphi consensus studies in parallel - one for meta-ethnography methodology experts and one for other stakeholders. In doing so we could differentiate between and include items of importance to either group. Consensus on an item was defined as ≥ 80% agreement that it was either “important” or “very important”. Items reaching this level of consensus in either eDelphi study would be included in the final reporting guidance.(28, 29)

#### Methods

##### Recruitment

###### Meta-ethnography methodology expert group

We aimed to purposively invite an international, multi-disciplinary panel of 45 methodological experts in qualitative evidence synthesis and meta-ethnography via professional networks, inviting authors of key texts identified in Stages 1 and 2, and using a snowballing approach. We anticipated a recruitment rate of 70% giving a final sample of at least 30. We defined a meta-ethnography expert participant as someone who met at least one of the following criteria:

- An academic with a reputation in qualitative evidence synthesis including, but not limited to, meta-ethnography.
- Author of a meta-ethnography or a methodological text in qualitative evidence synthesis or meta-ethnography considered by peers to be seminal.(3)

We emailed potential participants to invite them to participate. Ultimately, 71 potential *meta-ethnography expert* participants were invited to participate in the study of whom 48 individuals (68% recruitment rate) completed round 1 and 28 individuals (58% of those entering the study) completed three rounds of the study.

###### Key stakeholder expert group

We aimed to invite a diverse UK sample of approximately 45 key stakeholders comprise of 22-23 public/patient representatives and 22-23 professional evidence users. Ultimately, 48 *key stakeholder expert* participants were invited to participate in the study of whom 39 individuals completed round 1 and 23 individuals (59%) completed three rounds.

We defined a public/patient representative as someone who was aged ≥16 and met at least one of the following criteria:

- A member of the public or a patient or informal carer with an interest in health or social care research evidence
- A lay member of a clinical guideline development and/or funding panel.

Potential lay participants were identified and invited through voluntary and patient organisations, such as the Scottish Health Council, the Healthwatch and Public Involvement Association (HAPIA), and through the project team.

We defined a professional evidence user as someone who met at least one of the following criteria:

- Experience of producing reporting guidelines for other qualitative evidence synthesis approaches.
- Expertise in critical appraisal and evaluation of qualitative research studies.
- Editors and editorial board members of journals that publish meta-ethnographies and qualitative evidence syntheses e.g. Qualitative Health Research, Social Science and Medicine, Health Services Research.
- Worked for a government or non-government organisation that uses synthesised evidence on health/social care, or develops or disseminates evidence-based health/social care guidance and advice.
- Commissioned qualitative evidence syntheses.
- Worked in a role related to use of research evidence for health/social care policy or practice.
- Clinical guideline developer.
- Distilled evidence for policy makers.
- Health or social care policy maker.
- Used synthesised evidence or synthesises evidence in a professional non-academic capacity.(1)

Potential professional evidence-user participants were identified and invited through relevant organisations such as the Scottish Intercollegiate Guideline Network (SIGN), Healthcare Improvement Scotland (HIS), NICE, the Scottish Parliamentary Information Centre (SPICe), the International Guideline Network (G-I-N), and our existing networks.

##### Delphi Method

The Delphi method is a group consensus-reaching method(30) that presents questionnaires in a series of rounds, each one based on feedback from respondents’ responses to the previous questionnaire.(31) Participants are anonymous to each other, thus avoiding conformity to peer-group pressure and the design is suitable for administering to a geographically-dispersed panel ((p. 10).32)

##### eDelphi Procedure

We used a web-based platform developed for online ‘eDelphi’ studies at the University of Stirling. Rates of study participation are similar to paper-based administration methods ((p. 10).29, 32) The platform includes automated features such as the invitation by email, reminder and feedback processes. In each round, feedback on their own and the whole panel’s responses for each item were presented to participants visually as a colour histogram. This enabled participants to easily compare their responses to the consensus in the previous round and to then either confirm or update their response.

### Ethical approval

Ethical approval for the eDelphi study was granted from the University of Stirling School of Health Sciences Research Ethics Committee on 27/07/15.

### Data collection

Data collection took 12 weeks in total and comprised of three rounds, each lasting four weeks. Up to two electronic reminders were sent automatically to participants who had not yet completed the round. A set of 53 provisional items (relating to content) were presented in the first eDelphi round. Participants rated how important it was to them (on a four- point Likert-type scale 1= very unimportant, 4=very important) that the item should appear in the reporting guidance. Participants could record they had no expertise for any item listed. In Round 1 participants could add new items that they considered important (but none was suggested). In Rounds 2 and 3 they saw the same items they rated in the previous rounds and received feedback on the previous round: the relative frequency of responses for each item and their own responses.

### Analysis

Following completion of round three, frequencies and percentage of responses for each eDelphi study was calculated showing the level of consensus for each item. If an item reached consensus as being deemed important(33) or very important(34) in either eDelphi group it was included in the guidance.

**Results**

Most items (46/53) reached consensus (≥80% agreement that an item was important or very important) in both groups. Seven items did not reach consensus in the expert group and four items did not reach consensus for inclusion in both groups:-

- While acknowledging publication requirements and house style, the abstract should ideally: differentiate between reported findings of the primary studies and of the synthesis.
- State in which order primary study accounts had data extracted from them e.g. chronological or starting with an 'index' paper, and rationale for that order.
- State the order in which studies were translated/synthesised, e.g. chronologically from the earliest or most recent, and the rationale for this.
- State the qualitative research expertise of reviewers.

Therefore these four items were not included in the guidance.

The project team had to consider how the 49 items could be meaningfully presented in a usable format for end users of the guidance. Stage 4 of the project involved developing the guidance table and explanatory notes, developing training material and organising dissemination of the guidance.

# Stage 4 Guidance Development Process

There were too many items to form usable guidance in their eDelphi format. Moher *et al.* (2) provided a brief overview of the guidance development process following a consensus study but there was little literature to inform how to develop usable guidance from a large number of Delphi items such as generated in this project. We provide a summary here of the process we followed to develop the final reporting criteria and accompanying explanatory notes from the Delphi items. The guidance development process post-Delphi involved:

1. November 2016. Project Advisory Group Meeting (27 participants) - Refining the structure, content and nature of the reporting guidance
2. January 2017. Project Team Meeting - Merging items
3. February 2017. Project Advisory Group two Online Sessions (9 participants)- Usability of guidance
4. February-March 2017. Project Team Writing Group Sessions - Converting items into a guidance table, reporting criteria and explanatory notes
5. March 2017. Project Team Meeting - Refining the guidance table wording and style, and creating extensions
6. March-May 2017. Project Team and Project Advisory Group Co-Authors - Finalising the guidance table, reporting criteria, explanatory notes and extensions to the reporting criteria.(1)

Input from the Project Advisory Group at the 2016 meeting indicated that:

- guidance with too many items was unlikely to be used.
- a consistent level of detail should be given in the guidance table, with additional detail supplied in the accompanying explanatory notes.
- the guidance table should focus on what is key to good reporting, with suggestions of how this can be achieved described in the explanatory notes.
- the high level guidance should be relevant across disciplines and to a number of types of user, e.g. a meta-ethnography author, peer-reviewer, or an editor of a journal.

Therefore, a process was undergone, as listed above, through which items were reduced in number through merging items, restructuring items e.g. into Noblit and Hare’s 7 phases of meta-ethnography, moving detail of reporting requirements from the table of items/criteria to the explanatory notes, moving items into extensions to the guidance. Two levels of reporting were created - a high level summary of the reporting criteria for the guidance table, and the detailed explanatory notes that provided additional clarification.

The reporting criteria and explanatory notes were cross-checked against the items which had reached consensus in the Delphi studies (i) to check that no item had been missed from the re-writing process and (ii) to ensure that further detail had not been added to the guidance.

Three extensions to the guidance were created for reporting steps and processes that are not common to every meta-ethnography: (i) format and content of the meta-ethnography outputs e.g. title, abstract and keywords; (ii) assessment of methodological strengths and limitations of included primary studies e.g. quality appraisal; (iii) assessment of confidence in synthesised qualitative findings using GRADE CERQual (35, 36) Extensions (i) and (ii) were written from material removed from the guidance table and explanatory notes. Extension (iii) was written by a member of the project team (JN), who was involved in developing CERQual, in collaboration with the other CERQual originators. The final guidance table, explanatory notes and extensions were sent out for final feedback to the project team and Project Advisory Group members who qualified for authorship.

Following the process above, the number of items (criteria) in the final guidance reduced from **49** to **19**. A check was conducted of the detailed explanatory notes against the Stage 3 Delphi items which met consensus, to ensure that the meaning retained fidelity to the Delphi items.

# References

1. Cunningham M, France EF, Ring N, Uny I, Duncan EAS, Roberts RJ, et al. 13/114/60. Developing a reporting guideline to improve meta-ethnography in health research: the eMERGe mixed-methods study. Health Services and Delivery Research Journal. in press.

2. Moher D, Schulz KF, Simera I, Altman DG. Guidance for developers of health research reporting guidelines. PLoS Med. 2010;7(2):e1000217.

3. France EF, Ring N, Noyes J, Maxwell M, Jepson R, Duncan E, et al. Protocol-developing meta-ethnography reporting guidelines (eMERGe). BMC Med Res Methodol. 2015;15(1):103.

4. Dixon-Woods M, Booth A, Sutton AJ. Synthesizing qualitative research: a review of published reports. Qualitative Research. 2007;7(3):375-422.

5. France EF, Ring N, Thomas R, Noyes J, Maxwell M, Jepson R. A methodological systematic review of what's wrong with meta-ethnography reporting. BMC Med Res Methodol. 2014;14:119.

6. Hannes K, Macaitis K. A move to more systematic and transparent approaches in qualitative evidence synthesis: update on a review of published papers. Qualitative Research. 2012;12(4):402-42.

7. Q. S. R. International Pty Ltd. NVivo qualitative data analysis software. QSR International Pty Ltd;; 2012.

8. Noblit G, Hare D. Meta-Ethnography: Synthesiszing Qualitative Studies. California: Sage Publications; 1988. 88 p.

9. Kane GA, Wood VA, Barlow J. Parenting programmes: a systematic review and synthesis of qualitative research. Child Care Health Dev. 2007;33(6):784-93.

10. Ypinazar VA, Margolis SA, Haswell-Elkins M, Tsey K. Indigenous Australians' understandings regarding mental health and disorders. Aust N Z J Psychiatry. 2007;41(6):467-78.

11. Molony SL. The meaning of home: a qualitative meta-synthesis. Res Gerontol Nurs. 2010;3(4):291-307.

12. Purc-Stephenson RJ, Thrasher C. Nurses' experiences with telephone triage and advice: a meta-ethnography. J Adv Nurs. 2010;66(3):482-94.

13. Wikberg A, Bondas T. A patient perspective in research on intercultural caring in maternity care: A meta-ethnography. Int J Qual Stud Health Well-being. 2010;5(1).

14. Malterud K, Ulriksen K. Obesity, stigma, and responsibility in health care: A synthesis of qualitative studies. Int J Qual Stud Health Well-being. 2011;6(4).

15. Wells M, Williams B, Firnigl D, Lang H, Coyle J, Kroll T, et al. Returning to work after cancer: a qualitative meta-synthesis of problems, experiences and strategies of working after cancer. Chief Scientist’s Office: Edinburgh: 2011. Report CZG/2/467.

16. Garrett CR, Gask LL, Hays R, Cherrington A, Bundy C, Dickens C, et al. Accessing primary health care: a meta-ethnography of the experiences of British South Asian patients with diabetes, coronary heart disease or a mental health problem. Chronic Illn. 2012;8(2):135-55.

17. Hoy S. Beyond Men Behaving Badly: A Meta-Ethnography of Men's Perspectives on Psychological Distress and Help Seeking. International Journal of Men's Health. 2012;11(3):202-26.

18. Monforte-Royo C, Villavicencio-Chávez C, Tomás-Sábado J, Mahtani-Chugani V, Balaguer A. What lies behind the wish to hasten death? a systematic review and meta-ethnography from the perspective of patients. PLoS ONE. 2012;7(5):e37117.

19. Priddis H, Dahlen H, Schmied V. Women's experiences following severe perineal trauma: a meta-ethnographic synthesis. J Adv Nurs. 2013;69(4):748-59.

20. Sinnott C, Mc Hugh S, Browne J, Bradley C. GPs' perspectives on the management of patients with multimorbidity: systematic review and synthesis of qualitative research. BMJ Open. 2013;3(9):e003610.

21. Soundy A, Smith B, Dawes H, Pall H, Gimbrere K, Ramsay J. Patient's expression of hope and illness narratives in three neurological conditions: a meta-ethnography. Health Psychol Rev. 2013;7(2):177-201.

22. Wells M, Williams B, Firnigl D, Lang H, Coyle J, Kroll T, et al. Supporting 'work-related goals' rather than 'return to work' after cancer? A systematic review and meta-synthesis of 25 qualitative studies. Psychooncology. 2013;22(6):1208-19.

23. Cullinan S, O'Mahony D, Fleming A, Byrne S. A meta-synthesis of potentially inappropriate prescribing in older patients. Drugs Aging. 2014;31(8):631-8.

24. Hole E, Stubbs B, Roskell C, Soundy A. The Patient's Experience of the Psychosocial Process That Influences Identity following Stroke Rehabilitation: A Metaethnography. Scientific World Journal. 2014; Article ID 349151, 13 pages.

25. Errasti-Ibarrondo B, Perez M, Carrasco JM, Lama M, Zaragoza A, Arantzamendi M. Essential elements of the relationship between the nurse and the person with advanced and terminal cancer: A meta-ethnography. Nurs Outlook. 2015;63(3):255-68.

26. Galdas P, Darwin Z, Fell J, Kidd L, Bower P, Blickem C, et al. A systematic review and metaethnography to identify how effective, cost-effective, accessible and acceptable self-management support interventions are for men with long-term conditions (SELF-MAN). Health Services and Delivery Research. 2015; 3(34).

27. Lucas PJ, Cabral C, Hay AD, Horwood J. A systematic review of parent and clinician views and perceptions that influence prescribing decisions in relation to acute childhood infections in primary care. Scandinavian Journal of Primary Health Care. 2015;33(1):11-20.

28. Duncan E, Swingler K. A Web Site for Conducting Delphi Studies 2013. http://delphi.stir.ac.uk/about/ [Accessed 22 June 2018].

29. Duncan EA, Colver K, Dougall N, Swingler K, Stephenson J, Abhyankar P. Consensus on items and quantities of clinical equipment required to deal with a mass casualties big bang incident: a national Delphi study. BMC Emerg Med. 2014;14(1):5.

30. Linstone HA, Turoff M. The delphi method: techniques and applications. . Technometrics. 2002;18.

31. Hasson F, Keeney S, McKenna H. Research guidelines for the Delphi survey technique. J Adv Nurs. 2000;32(4):1008-15.

32. France EF, Ring N, Noyes J, Maxwell M, Jepson R, Duncan E, et al. Protocol-developing meta-ethnography reporting guidelines (eMERGe). BMC Med Res Methodol. 2015;15(1):103.

33. Noyes JL, S.;. Chapter 6: Supplemental Guidance on Selecting a Method of Qualitative Evidence Synthesis, and Integrating Qualitative Evidence with Cochrane Intervention Reviews. . In: Noyes J BA, Hannes K, Harden A, Harris J, Lewin S, Lockwood C., editor. Supplementary Guidance for Inclusion of Qualitative Research in Cochrane Systematic Reviews of Interventions Version 1 (updated August 2011) Cochrane Collaboration Qualitative Methods Group; 2011.

34. Atkins S, Lewin S, Smith H, Engel M, Fretheim A, Volmink J. Conducting a meta-ethnography of qualitative literature: lessons learnt. BMC Med Res Methodol. 2008;8:21.

35. Lewin S, Glenton C, Munthe-Kaas H, Carlsen B, Colvin CJ, Gulmezoglu M, et al. Using qualitative evidence in decision making for health and social interventions: an approach to assess confidence in findings from qualitative evidence syntheses (GRADE-CERQual). PLoS Med. 2015;12(10):e1001895.

36. Noyes J, Booth A, Flemming K, Garside R, Harden A, Lewin S, et al. Cochrane Qualitative and Implementation Methods Group guidance series-paper 3: methods for assessing methodological limitations, data extraction and synthesis, and confidence in synthesized qualitative findings. Journal of clinical epidemiology. 2018;97:49-58.

## Appendix 1. PRISMA flow diagram for Stage 1

## Identification

Records identified through advanced database searching
(n = 9285)

## Screening

## Included

## Eligibility

Records identified from other sources (*e.g. experts, citations pearl-searching*)
(n = 47)

Records screened
(n = 7522)

Records excluded
(n = 7417)

Full-text articles assessed for eligibility
(n = 105)

Full-text articles excluded at data extraction

(n = 48)

*Reasons: clearly irrelevant once full-text obtained ; did not report methodological issues about meta-ethnography; not a reporting guideline nor providing guidance on reporting of meta-ethnography*

Studies included in the Review
**(n = 57)**

Records after duplicates removed
(n = 7522)

Copyright statement: this PRISMA diagram contains public sector information licensed under the Open Government Licence v3.0.

Adapted From: Moher D, Liberati A, Tetzlaff J, Altman DG, The PRISMA Group (2009). Preferred Reporting Items for Systematic Reviews and Meta-Analyses: The PRISMA Statement. PLoS Med 6(6): e1000097. doi:10.1371/journal.pmed1000097

## Appendix 2. PRISMA adapted flow diagram for Stage 2.2

Records identified through bibliographic database searching (n=1080)

## Screening

## Sampling

## Eligibility

## Identification

Records screened by title & abstract
(n **=** 571)

Records meeting purposive sampling inclusion criteria (n **=** 243)

Purposive audit sample of publications labelled as meta-ethnographies
(n =40)

## Included

Meta-ethnographies in final purposive audit sample
**(n =19)**

Records excluded (n = 328)

Additional records identified through other sources (n=420)

Records after duplicates removed (n **=**571)

Items labelled as meta-ethnographies, but judged to be other qualitative evidence synthesis design during audit (n =21)

|  |
| --- |

Copyright statement: this PRISMA diagram contains public sector information licensed under the Open Government Licence v3.0.

*From: Moher D, Liberati A, Tetzlaff J, Altman DG, The PRISMA Group (2009). Preferred Reporting Items for Systematic Reviews and Meta-Analyses: The PRISMA Statement. PLoS Med 6(6): e1000097. doi:10.1371/journal.pmed1000097*
