# Supplementary material for: Improving reporting of meta-ethnography: the eMERGe reporting guidance
Source: BMC Med Res Methodol. 2019 Jan 31;19:25. doi: 10.1186/s12874-018-0600-0 (PMC6359764; doi:10.1186/s12874-018-0600-0)
Supplement: Supplementary file 2 — File S2. Supplementary information: A. 57 publications included in the systematic review for ‘Stage1 Identification of Standards’and B. Publications contributing to development of reporting criteria. (DOCX 299 kb) [file 12874_2018_600_MOESM2_ESM.docx]

File S2. Supplementary information: A. 57 publications included in the systematic review for ‘Stage 1 Identification of Standards’ and B. Publications contributing to development of reporting criteria

**A. Publications included in the methodological systematic review**

1. ATKINS, S., LEWIN, S., SMITH, H., ENGEL, M., FRETHEIM, A. & VOLMINK, J. 2008. Conducting a meta-ethnography of qualitative literature: lessons learnt. *BMC Med Res Methodol,* 8**,** 21.
2. BARNETT-PAGE, E. & THOMAS, J. 2009. Methods for the synthesis of qualitative research: a critical review. *BMC Med Res Methodol,* 9**,** 59.
3. BEARMAN, M. & DAWSON, P. 2013. Qualitative synthesis and systematic review in health professions education. *Med Educ,* 47**,** 252-60.
4. BECK, C. T. 2009. Metasynthesis: a goldmine for evidence-based practice. *AORN J,* 90**,** 701-2, 705-10.
5. BONDAS, T. & HALL, E. O. 2007a. Challenges in approaching metasynthesis research. *Qual Health Res,* 17**,** 113-21.
6. BONDAS, T. & HALL, E. O. C. 2007b. A decade of metasynthesis research in health sciences: A meta-method study. *International Journal of Qualitative Studies on Health and Well-being,* 2**,** 101-113.
7. BOOTH, A. 2013. *Acknowledging a Dual Heritage for Qualitative Evidence Synthesis: Harnessing the Qualitative Research and Systematic Review Research Traditions* PhD, University of Sheffield. .
8. BOOTH, A., CARROLL, C., ILOTT, I., LOW, L. L. & COOPER, K. 2013. Desperately seeking dissonance: identifying the disconfirming case in qualitative evidence synthesis. *Qual Health Res,* 23**,** 126-41.
9. BOOTH, A., NOYES, J., FLEMMING, K., GERHARDUS, A., WAHLSTER, P., VAN DER WILT, G., MOZYGEMBA, K., REFOLO, P., SACCHINI, D., TUMMERS, M. & REHFUESS, E. 2016. Guidance on choosing qualitative evidence synthesis methods for use in health technology assessments of complex interventions. INTEGRATE-HTA.
10. BRITTEN, N., CAMPBELL, R., POPE, C., DONOVAN, J., MORGAN, M. & PILL, R. 2002. Using meta ethnography to synthesise qualitative research: a worked example. *Journal of Health Services & Research Policy,* 7**,** 209-215.
11. BRITTEN, N. & POPE, C. 2012. Medicine taking for asthma: a worked example of meta-ethnography (Chapter 3). *In:* HANNES, K. & LOCKWOOD, C. (eds.). Chichester: Wiley-Blackwell BMJ Books.
12. CAMPBELL, R., BRITTEN, N., POUND, P., DONOVAN, J., MORGAN, M., PILL, R. & POPE, C. 2006 Section 4.8- Using meta-ethnography to synthesise qualitative research. *In:* POPAY, J. (ed.) *Moving beyond effectiveness in evidence synthesis: Methodological issues in the synthesis of diverse sources of evidence.* London: NICE.
13. CAMPBELL, R., POUND, P., MORGAN, M., DAKER-WHITE, G., BRITTEN, N., PILL, R., YARDLEY, L., POPE, C. & DONOVAN, J. 2011. Evaluating meta-ethnography: systematic analysis and synthesis of qualitative research. *Health Technol Assess,* 15**,** 1-164.
14. CAMPBELL, R., POUND, P., POPE, C., BRITTEN, N., PILL, R., MORGAN, M. & DONOVAN, J. 2003. Evaluating meta-ethnography: a synthesis of qualitative research on lay experiences of diabetes and diabetes care. *Soc Sci Med,* 56**,** 671-84.
15. CARROLL, C. & BOOTH, A. 2015. Quality assessment of qualitative evidence for systematic review and synthesis: Is it meaningful, and if so, how should it be performed? *Res Synth Methods,* 6**,** 149-54.
16. DIXON-WOODS, M., AGARWAL, S., JONES, D., YOUNG, B. & SUTTON, A. 2005. Synthesising qualitative and quantitative evidence: a review of possible methods. *J Health Serv Res Policy,* 10**,** 45-53.
17. DIXON-WOODS, M., AGARWAL, S., YOUNG, B., JONES, D. & SUTTON, A. J. 2004. Integrative approaches to qualitative and quantitative evidence. *NHS Health Development Agency.*
18. DIXON-WOODS, M., BOOTH, A. & SUTTON, A. J. 2007. Synthesizing qualitative research: a review of published reports. *Qualitative Research,* 7**,** 375-422.
19. DOYLE, L. H. 2003. Synthesis through meta-ethnography: paradoxes, enhancements, and possibilities. *Qualitative Research,* 3**,** 321-344.
20. ERASMUS, E. 2014. The use of street-level bureaucracy theory in health policy analysis in low- and middle-income countries: a meta-ethnographic synthesis. *Health Policy Plan,* 29 Suppl 3**,** iii70-8.
21. FINFGELD-CONNETT, D. 2014. Metasynthesis findings: potential versus reality. *Qual Health Res,* 24**,** 1581-91.
22. FINFGELD-CONNETT, D. & JOHNSON, E. D. 2013. Literature search strategies for conducting knowledge-building and theory-generating qualitative systematic reviews. *J Adv Nurs,* 69**,** 194-204.
23. FINLAYSON, K. W. & DIXON, A. 2008. Qualitative meta-synthesis: a guide for the novice. *Nurse Res,* 15**,** 59-71.
24. FRANCE, E. F., RING, N., THOMAS, R., NOYES, J., MAXWELL, M. & JEPSON, R. 2014. A methodological systematic review of what's wrong with meta-ethnography reporting. *BMC Med Res Methodol,* 14**,** 119.
25. FRANCE, E. F. W., M.; LANG, H.; WILLIAMS, B.; 2016. Why, when and how to update a meta-ethnography qualitative synthesis. *Systematic reviews,* 5**,** 44.
26. FRANZEL, B., SCHWIEGERSHAUSEN, M., HEUSSER, P. & BERGER, B. 2013. How to locate and appraise qualitative research in complementary and alternative medicine. *BMC Complement Altern Med,* 13**,** 125.
27. GARSIDE, R. 2008. *A Comparison of methods for the Systematic Review of Qualitative Research : Two Examples Using Meta-Ethnography and Meta-Study.* PhD, University of Exeter.
28. HAMMERSLEY, M. 2013. Chapter 11- What is qualitative synthesis and why we do it? *The myth of research based policy.* Sage.
29. HANNES, K. & MACAITIS, K. 2012. A move to more systematic and transparent approaches in qualitative evidence synthesis: update on a review of published papers. *Qualitative Research,* 12**,** 402-442.
30. HANSEN, H. P., DRABORG, E. & KRISTENSEN, F. B. 2011. Exploring qualitative research synthesis: the role of patients' perspectives in health policy design and decision making. *Patient,* 4**,** 143-52.
31. KANGASNIEMI, M., LANSIMIES-ANTIKAINEN, H., HALKOAHO, A. & PIETILA, A. M. 2012. Examination of the phases of metasynthesis: a study on patients' duties as an example. *Prof Inferm,* 65**,** 55-60.
32. KINN, L. G., HOLGERSEN, H., EKELAND, T. J. & DAVIDSON, L. 2013. Metasynthesis and bricolage: an artistic exercise of creating a collage of meaning. *Qual Health Res,* 23**,** 1285-92.
33. LEE, R. P., HART, R. I., WATSON, R. M. & RAPLEY, T. 2015. Qualitative synthesis in practice: some pragmatics of meta-ethnography. *Qualitative Research,* 15**,** 334-350.
34. MALPASS, A., SHAW, A., SHARP, D., WALTER, F., FEDER, G., RIDD, M. & KESSLER, D. 2009. "Medication career" or "moral career"? The two sides of managing antidepressants: a meta-ethnography of patients' experience of antidepressants. *Soc Sci Med,* 68**,** 154-68.
35. MCCANN, S., CAMPBELL, M. & ENTWISTLE, V. 2013. Recruitment to clinical trials: a meta-ethnographic synthesis of studies of reasons for participation. *J Health Serv Res Policy,* 18**,** 233-41.
36. MCCORMICK, J., RODNEY, P. & VARCOE, C. 2003. Reinterpretations across studies: an approach to meta-analysis. *Qual Health Res,* 13**,** 933-44.
37. MEADOWS-OLIVER, M. 2015. Meta-ethnography. *In:* DE CHESNAY, M. D. C., MARY (ed.) *Nursing research using ethnography: Qualitative designs and methods in nursing.* New York, NY, US: Springer Publishing Co.
38. MELENDEZ-TORRES, G. J., GRANT, S. & BONELL, C. 2015. A systematic review and critical appraisal of qualitative metasynthetic practice in public health to develop a taxonomy of operations of reciprocal translation. *Res Synth Methods,* 6**,** 357-71.
39. NOBLIT, G. W. & HARE, R. D. 1988. *Meta-Ethnography: Synthesizing Qualitative Studies,* California, Sage Publications.
40. NOYES, J. & LEWIN, S. 2011. Chapter 6: Supplemental Guidance on Selecting a Method of Qualitative Evidence Synthesis, and Integrating Qualitative Evidence with Cochrane Intervention Reviews. . *In:* NOYES J., BOOTH A., HANNES K., HARDEN A., HARRIS J., LEWIN S. & C., L. (eds.) *Supplementary Guidance for Inclusion of Qualitative Research in Cochrane Systematic Reviews of Interventions. Version 1 (updated August 2011). .* Cochrane Collaboration Qualitative Methods Group.
41. NYE, E., MELENDEZ-TORRES, G. J. & BONNELL, C. 2016. Origins, methods, and advances in qualitative meta-synthesis. *Review of Education,* 4**,** 57-79.
42. PATERSON, B. L. 2011. “It Looks Great but How do I know if it Fits?”: An Introduction to Meta-Synthesis Research. *Synthesizing Qualitative Research.* John Wiley & Sons, Ltd.
43. POPE, C. & MAYS, N. 2006. Synthesising qualitative research. *In:* POPE, C. & MAYS, N. (eds.) *Qualitative research in health care (3rd ed.).* Oxford UK: Blackwell Publishing;BMJ Books.
44. POPE, C., MAYS, N. & POPAY, J. 2007. Chapter 4- Interpretive approaches to evidence synthesis. *Synthesizing qualitative and quantitative health evidence: a guide to methods.*: Open University Press.
45. RING, N., JEPSON, R. & RITCHIE, K. 2011. Methods of synthesizing qualitative research studies for health technology assessment. *Int J Technol Assess Health Care,* 27**,** 384-90.
46. RING, N., RITCHIE, K., MANDAVA, L. & JEPSON, R. 2010. A guide to synthesising qualitative research for researchers undertaking health technology assessments and systematic reviews *Health Technology Assessment Database.* NHS Quality Improvement Scotland (NHS QIS).
47. SAINI, M., & SHLONSKY, A. 2012. *Systematic synthesis of qualitative research.*, Oxford University Press.
48. SEERS, K. 2015. Qualitative systematic reviews: their importance for our understanding of research relevant to pain. *Br J Pain,* 9**,** 36-40.
49. SIGURDSON, C. & WOODGATE, R. 2015. Designing a Metasynthesis Study in Pediatric Oncology Nursing Research. *J Pediatr Oncol Nurs,* 32**,** 360-8.
50. SURI, H. & CLARKE, D. 2009. Advancements in Research Synthesis Methods: From a Methodologically Inclusive Perspective. *Review of Educational Research,* 79**,** 395-430.
51. THORNE, S., JENSEN, L., KEARNEY, M. H., NOBLIT, G. & SANDELOWSKI, M. 2004. Qualitative metasynthesis: reflections on methodological orientation and ideological agenda. *Qual Health Res,* 14**,** 1342-65.
52. TONG, A., FLEMMING, K., MCINNES, E., OLIVER, S. & CRAIG, J. 2012. Enhancing transparency in reporting the synthesis of qualitative research: ENTREQ. *BMC Med Res Methodol,* 12**,** 181.
53. TOYE, F., SEERS, K., ALLCOCK, N., BRIGGS, M., CARR, E., ANDREWS, J. & BARKER, K. 2013. 'Trying to pin down jelly' - exploring intuitive processes in quality assessment for meta-ethnography. *Bmc Medical Research Methodology,* 13**,** 46.
54. TOYE, F., SEERS, K., ALLCOCK, N., BRIGGS, M., CARR, E. & BARKER, K. 2014. Meta-ethnography 25 years on: challenges and insights for synthesising a large number of qualitative studies. *BMC Med Res Methodol,* 14**,** 80.
55. WALSH, D. & DOWNE, S. 2005. Meta-synthesis method for qualitative research: a literature review. *J Adv Nurs,* 50**,** 204-11.
56. WEED, M. 2006. Interpretive qualitative synthesis in the sport & exercise sciences: The meta-interpretation approach. *European Journal of Sport Science,* 6**,** 127-139.
57. WEED, M. 2008. A Potential Method for the Interpretive Synthesis of Qualitative Research: Issues in the Development of 'Meta-Interpretation'. *International Journal of Social Research Methodology,* 11**,** 13-28.

##

## B. Methodological publications contributing to development of reporting criteria

| Aspect / Phase of meta-ethnography | Publications contributing relevant data/ evidence |
| --- | --- |
| Nature of meta-ethnography and how it differs from other qualitative evidence synthesis methodologies | (Atkins et al., 2008, Bondas and Hall, 2007b, Bondas and Hall, 2007a, Booth, 2001, Britten et al., 2002, Campbell et al., 2003, Dixon-Woods et al., 2004, Dixon-Woods et al., 2005, Doyle, 2003, Malpass et al., 2009, Noblit and Hare, 1988, Pope and Mays, 2006, Barnett-Page and Thomas, 2009, Bearman and Dawson, 2013, Beck, 2009, Booth, 2013, Booth et al., 2016, Britten and Pope, 2012, Campbell et al., 2011, Toye et al., 2014) |
| Selecting a qualitative evidence synthesis methodology | (Atkins et al., 2008, Bearman and Dawson, 2013, Beck, 2009, Britten et al., 2002, Campbell et al., 2003, Campbell et al., 2011, Finlayson and Dixon, 2008, Hannes and Macaitis, 2012, Malpass et al., 2009, Noyes and Lewin, 2011, Paterson, 2011, Suri and Clarke, 2009, Tong et al., 2012, Booth, 2013, Toye et al., 2014, Meadows-Oliver, 2015, Melendez-Torres et al., 2015, Garside, 2008) |
| Phase 1- Getting Started | (Atkins et al., 2008, Booth, 2013, Britten et al., 2002, Campbell et al., 2003, Dixon-Woods et al., 2005, Finlayson and Dixon, 2008, Noblit and Hare, 1988, Garside, 2008, Booth et al., 2016, Campbell et al., 2011, Finfgeld-Connett, 2014, Finfgeld-Connett and Johnson, 2013, Kangasniemi et al., 2012, Kinn et al., 2013, Meadows-Oliver, 2015, Sigurdson and Woodgate, 2015, Toye et al., 2014) |
| Phase 2 – Deciding what is relevant | (Atkins et al., 2008, Britten and Pope, 2012, Britten et al., 2002, Campbell et al., 2011, Dixon-Woods et al., 2005, Finlayson and Dixon, 2008, Garside, 2008, Hannes and Macaitis, 2012, Kangasniemi et al., 2012, Noblit and Hare, 1988, Suri and Clarke, 2009, Tong et al., 2012, Walsh and Downe, 2005, Weed, 2008, Booth, 2013, Booth et al., 2013, Carroll and Booth, 2015, Finfgeld-Connett, 2014, Finfgeld-Connett and Johnson, 2013, France et al., 2014, Kinn et al., 2013, Meadows-Oliver, 2015, Nye et al., 2016, Toye et al., 2013, Toye et al., 2014) |
| Phase 3- Reading Studies | (Atkins et al., 2008, Bondas and Hall, 2007a, Booth, 2013, Britten et al., 2002, Campbell et al., 2006 , Campbell et al., 2003, Kangasniemi et al., 2012, Toye et al., 2014, Noblit and Hare, 1988, Campbell et al., 2011, Erasmus, 2014, Malpass et al., 2009, Sigurdson and Woodgate, 2015, Lee et al., 2015, France et al., 2014, Garside et al., 2008) |
| Phase 4- Determining how the studies are related | (Britten and Pope, 2012, Campbell et al., 2011, Erasmus, 2014, Malpass et al., 2009, Atkins et al., 2008, Britten et al., 2002, Campbell et al., 2003, Beck, 2009, Booth et al., 2013, Doyle, 2003, Toye et al., 2014, Booth et al., 2016, France et al., 2014, Noblit and Hare, 1988) |
| Phase 5- Translating Studies into one another | (Booth, 2013, Campbell et al., 2006 , Campbell et al., 2003, Garside, 2008, Atkins et al., 2008, Campbell et al., 2011, Doyle, 2003, Erasmus, 2014, Toye et al., 2014, Lee et al., 2015, Britten et al., 2002, Finfgeld-Connett, 2014, Malpass et al., 2009, Melendez-Torres et al., 2015, Thorne et al., 2004, Weed, 2008, Barnett-Page and Thomas, 2009, Bondas and Hall, 2007a, Booth et al., 2013, Britten and Pope, 2012, Kinn et al., 2013, Noblit and Hare, 1988, Pope and Mays, 2006, Walsh and Downe, 2005, Suri and Clarke, 2009, Booth et al., 2016, McCann et al., 2013, Dixon-Woods et al., 2005) |
| Phase 6- Synthesising translations | (Britten et al., 2002, Doyle, 2003, Noblit and Hare, 1988, Atkins et al., 2008, Booth, 2013, Campbell et al., 2011, Thorne et al., 2004, Britten and Pope, 2012, Bondas and Hall, 2007a, Campbell et al., 2003, Finfgeld-Connett, 2014) |
| Phase 7 - Expressing the Synthesis | (Doyle, 2003, Noblit and Hare, 1988, Atkins et al., 2008, Britten et al., 2002, Bondas and Hall, 2007a, Bearman and Dawson, 2013, Beck, 2009, Campbell et al., 2006 , Campbell et al., 2011, Hannes and Macaitis, 2012, Pope et al., 2007, Toye et al., 2014, Booth, 2013) |
| Issues of primary study context in meta-ethnography | (Atkins et al., 2008, Campbell et al., 2011, Noblit and Hare, 1988, Thorne et al., 2004, Booth, 2013, Britten et al., 2002, Toye et al., 2013, Toye et al., 2014) |
| Number of reviewers required to undertake a meta-ethnography | (Atkins et al., 2008, Bearman and Dawson, 2013, Booth et al., 2013, Erasmus, 2014, France et al., 2014, Garside, 2008, Lee et al., 2015, McCormick et al., 2003, Walsh and Downe, 2005, Bondas and Hall, 2007b, Booth, 2013, Finlayson and Dixon, 2008, Kangasniemi et al., 2012, Toye et al., 2014, Sigurdson and Woodgate, 2015, Bondas and Hall, 2007a, Campbell et al., 2011) |
| Validity, credibility and transferability issues in meta-ethnography | (Noblit and Hare, 1988, Campbell et al., 2011, Hammersley, 2013, Booth et al., 2013, Doyle, 2003, McCormick et al., 2003, Campbell et al., 2003, Garside, 2008, Melendez-Torres et al., 2015, Thorne et al., 2004, Bondas and Hall, 2007a, Booth, 2013, Britten et al., 2002, Lee et al., 2015, Meadows-Oliver, 2015, Campbell et al., 2006 , Dixon-Woods et al., 2004, Finfgeld-Connett and Johnson, 2013, Kinn et al., 2013) |
