# Supplementary material for: Improving reporting of meta-ethnography: the eMERGe reporting guidance
Source: BMC Med Res Methodol. 2019 Jan 31;19:25. doi: 10.1186/s12874-018-0600-0 (PMC6359764; doi:10.1186/s12874-018-0600-0)
Supplement: Supplementary file 3 — File S3. Supplementary information: the sample of 29 published meta-ethnographies analysed in ‘Stage 2 Development and Application of Standards’. (DOCX 27 kb) [file 12874_2018_600_MOESM3_ESM.docx]

File S3. Supplementary information: the sample of 29 published meta-ethnographies analysed in ‘Stage 2 Development and Application of Standards’

**Seminal meta-ethnographies**

1. Ayar MC, Bauchspies WK, Yalvac B. Examining Interpretive Studies of Science: A Meta-ethnography. *Educational Sciences-Theory & Practice* 2015;**15**:253-65. <http://dx.doi.org/10.12738/estp.2015.1.2153>

2. Beach D, Bagley C, Eriksson A, Player-Koro C. Changing teacher education in Sweden: Using meta-ethnographic analysis to understand and describe policy making and educational changes. *Teaching and Teacher Education* 2014;**44**:160-7. <http://dx.doi.org/10.1016/j.tate.2014.08.011>

3. Britten N, Campbell R, Pope C, Donovan J, Morgan M, Pill R. Using meta ethnography to synthesise qualitative research: a worked example. *Journal of Health Services & Research Policy* 2002;**7**:209-15.

4. Britten N, Pope C. Medicine taking for asthma: a worked example of meta-ethnography (Chapter 3). In: Hannes K, Lockwood C, editors.Chichester: Wiley-Blackwell BMJ Books; 2012:41-58.

5. Campbell R, Pound P, Morgan M, Daker-White G, Britten N, Pill R*, et al.* Evaluating meta-ethnography: systematic analysis and synthesis of qualitative research. *Health Technol Assess* 2011;**15**:1-164. <http://dx.doi.org/10.3310/hta15430>

6. Campbell R, Pound P, Pope C, Britten N, Pill R, Morgan M*, et al.* Evaluating meta-ethnography: a synthesis of qualitative research on lay experiences of diabetes and diabetes care. *Soc Sci Med* 2003;**56**:671-84.

7. Garside R, Britten N, Stein K. The experience of heavy menstrual bleeding: a systematic review and meta-ethnography of qualitative studies. *J Adv Nurs* 2008;**63**:550-62. <http://dx.doi.org/10.1111/j.1365-2648.2008.04750.x>

8. Gomersall T, Madill A, Summers LK. A metasynthesis of the self-management of type 2 diabetes. *Qual Health Res* 2011;**21**:853-71. ^[[1]](#footnote-1)^<http://dx.doi.org/10.1177/1049732311402096>

9. Malpass A, Shaw A, Sharp D, Walter F, Feder G, Ridd M*, et al.* "Medication career" or "moral career"? The two sides of managing antidepressants: a meta-ethnography of patients' experience of antidepressants. *Soc Sci Med* 2009;**68**:154-68. <http://dx.doi.org/10.1016/j.socscimed.2008.09.068>

10. Munro SA, Lewin SA, Smith HJ, Engel ME, Fretheim A, Volmink J. Patient adherence to tuberculosis treatment: a systematic review of qualitative research. *PLoS Med* 2007;**4**:e238. <http://dx.doi.org/10.1371/journal.pmed.0040238>

11. Pound P, Britten N, Morgan M, Yardley L, Pope C, Daker-White G*, et al.* Resisting medicines: a synthesis of qualitative studies of medicine taking. *Soc Sci Med* 2005;**61**:133-55. <http://dx.doi.org/10.1016/j.socscimed.2004.11.063>

12. Toye F, Seers K, Allcock N, Briggs M, Carr E, Andrews J*, et al.* Patients' experiences of chronic non-malignant musculoskeletal pain: a qualitative systematic review. *Br J Gen Pract* 2013;**63**:e829-41. <http://dx.doi.org/10.3399/bjgp13X675412>

13. Vittner D, Casavant S, McGrath JM. A Meta-ethnography: Skin-to-Skin Holding From the Caregiver's Perspective. *Adv Neonatal Care* 2015;**15**:191-200; quiz E1-2. <http://dx.doi.org/10.1097/ANC.0000000000000169>

**Relatively poorly reported meta-ethnographies**

14. Brohan E, Henderson C, Wheat K, Malcolm E, Clement S, Barley EA*, et al.* Systematic review of beliefs, behaviours and influencing factors associated with disclosure of a mental health problem in the workplace. *BMC Psychiatry* 2012;**12**:11. <http://dx.doi.org/10.1186/1471-244X-12-11>

15. Cairns V, Murray C. How do the features of mindfulness-based cognitive therapy contribute to positive therapeutic change? A meta-synthesis of qualitative studies. *BehavCogn Psychother* 2015;**43**:342-59.

16. Child S, Goodwin V, Garside R, Jones-Hughes T, Boddy K, Stein K. Factors influencing the implementation of fall-prevention programmes: a systematic review and synthesis of qualitative studies. *Implement Sci* 2012;**7**:91. <http://dx.doi.org/10.1186/1748-5908-7-91>

17. Furuta M, Sandall J, Bick D. Women's perceptions and experiences of severe maternal morbidity--a synthesis of qualitative studies using a meta-ethnographic approach. *Midwifery* 2014;**30**:158-69. <http://dx.doi.org/10.1016/j.midw.2013.09.001>

18. Jensen LA, Allen MN. A Synthesis of Qualitative Research on Wellness-Illness. *Qualitative Health Research* 1994;**4**:349-69. <http://dx.doi.org/10.1177/104973239400400402>

19. Lundgren I, Begley C, Gross MM, Bondas T. 'Groping through the fog': a metasynthesis of women's experiences on VBAC (Vaginal birth after Caesarean section). *BMC Pregnancy Childbirth* 2012;**12**:85. <http://dx.doi.org/10.1186/1471-2393-12-85>

20. Nelson AM. A meta-synthesis related to infant feeding decision making. *MCN Am J Matern Child Nurs* 2012;**37**:247-52. <http://dx.doi.org/10.1097/NMC.0b013e31824fde7d>

21. O'Neill T, Jinks C, Ong BN. Decision-making regarding total knee replacement surgery: A qualitative meta-synthesis. *Bmc Health Services Research* 2007;**7**:52-. <http://dx.doi.org/Artn> 52

10.1186/1472-6963-7-52

22. Rudolfsson G, Berggren I. Nursing students' perspectives on the patient and the impact of the nursing culture: a meta-synthesis. *J Nurs Manag* 2012;**20**:771-81. <http://dx.doi.org/10.1111/j.1365-2834.2012.01470.x>

23. Schmied V, Olley H, Burns E, Duff M, Dennis CL, Dahlen HG. Contradictions and conflict: a meta-ethnographic study of migrant women's experiences of breastfeeding in a new country. *BMC Pregnancy Childbirth* 2012;**12**:163. <http://dx.doi.org/10.1186/1471-2393-12-163>

24. Smith LK, Pope C, Botha JL. Patients' help-seeking experiences and delay in cancer presentation: a qualitative synthesis. *Lancet* 2005;**366**:825-31. <http://dx.doi.org/10.1016/S0140-6736(05)67030-4>

25. Smith TO, Purdy R, Lister S, Salter C, Fleetcroft R, Conaghan PG. Attitudes of people with osteoarthritis towards their conservative management: a systematic review and meta-ethnography. *Rheumatology International* 2014;**34**:299-313. <http://dx.doi.org/10.1007/s00296-013-2905-y>

26. Steen M, Downe S, Bamford N, Edozien L. Not-patient and not-visitor: a metasynthesis fathers' encounters with pregnancy, birth and maternity care. *Midwifery* 2012;**28**:362-71. <http://dx.doi.org/10.1016/j.midw.2011.06.009>

27. Thorne S, Paterson B. Shifting images of chronic illness. *Image J Nurs Sch* 1998;**30**:173-8.

28. Tuthill E, McGrath J, Young S. Commonalities and differences in infant feeding attitudes and practices in the context of HIV in sub-Saharan Africa: a metasynthesis. *AIDS Care* 2014;**26**:214-25. <http://dx.doi.org/10.1080/09540121.2013.813625>

29. Tuquero JM. A Meta-Ethnographic Synthesis of Support Services in Distance Learning Programs. *Journal of Information Technology Education*

2011;**10**:IIP 157-IIP 79.

1. Gomersall et al was recommended as an example of a high-quality qualitative evidence synthesis that drew on a range of synthesis methodologies, not just meta-ethnography. [↑](#footnote-ref-1)
