# Supplementary material for: Improving reporting of meta-ethnography: the eMERGe reporting guidance
Source: BMC Med Res Methodol. 2019 Jan 31;19:25. doi: 10.1186/s12874-018-0600-0 (PMC6359764; doi:10.1186/s12874-018-0600-0)
Supplement: Supplementary file 4 — Table S4. Supplementary information: explanatory notes for Phases 3-6 to accompany Part 2 of the guidance. (DOCX 88 kb) [file 12874_2018_600_MOESM4_ESM.docx]

Table S4. Supplementary information: explanatory notes for Phases 3-6 to accompany Part 2 of the guidance

| **No.** | **Criteria Headings** | | **Supplementary data** |
| --- | --- | --- | --- |
| **Phase 3 – Reading included studies** | | | |
| 9 | | Reading and data extraction approach | The systematic review findings in Stage 1 of the eMERGe project indicated that reading is not a discrete phase in meta-ethnography conduct (Noblit and Hare 1988, Toye et al. 2014). Reading is usually combined with identifying and recording primary study concepts (or metaphors or themes) and their context, e.g. (Atkins et al. 2008, Bondas and Hall 2007, Booth 2013, Britten et al. 2002, Kangasniemi et al. 2012), and has also been combined with quality appraisal of studies (Campbell et al. 2011) and judging the suitability of studies for inclusion in the meta-ethnography (Kangasniemi et al. 2012, Lee et al. 2015).  There is currently no agreed, standardised terminology for some of the meta-ethnography analytical and synthesis processes. For example, a range of terms, such as themes, metaphors, or concepts, has been used for the conceptual data in primary studies by different reviewers. Reviewers should more clearly define their terminology to aid the reader’s understanding of the methodological processes (France et al. 2014). |
| 10 | | Presenting characteristics of included studies | Meta-ethnography was designed specifically to preserve the contextual aspects of studies included in a synthesis because context is important to data interpretation (Noblit and Hare 1988). Noblit and Hare (1988) have contended that aggregative qualitative evidence syntheses were ‘context-stripping [and] impeded explanation and thus negated a true interpretive synthesis’ (Noblit and Hare 1988, p.23). This is why it is important for reviewers to describe the context of each included primary study (Atkins et al. 2008, Thorne et al. 2004), where those data are provided (context is often poorly reported in primary studies). |

| **Phase 4 – Determining how studies are related** | | |
| --- | --- | --- |
| 11 | Process for determining how studies are related | A common weakness in published meta-ethnographies is reviewers not describing if or how they determined how included studies are related (France et al. 2014).  Noblit and Hare (1988) stated that primary studies may relate to one another in three main ways:   - reciprocally (because they are about similar things), - refutationally (because they contradict one another) - or as a line of argument (because they are about different aspects of the topic being studied).   Concepts from studies, the findings, and/or research paradigms and theoretical approaches adopted may relate to each other reciprocally or refutationally (Bondas and Hall 2007, Britten and Pope 2012, Finfgeld-Connett 2014, Noblit and Hare 1988).  One example of a method for comparing studies is to juxtapose concepts from the primary studies in a grid in order to identify the relationship between them (Campbell et al. 2011). The way in which studies or concepts are related influences how the translation (Phase 5) is conducted. |
| 12 | Outcome of relating studies | Some authors of worked examples of meta-ethnographies have shown how they related the studies in a grid or table (Britten and Pope 2012, Erasmus 2014, Malpass et al. 2009). |

| **Phase 5 – Translating studies into one another** | | |
| --- | --- | --- |
| 13 | Process of translating studies | Our systematic review identified that translation is key, and possibly unique, to meta-ethnography compared to other qualitative evidence synthesis methodologies. Translations are not literal but idiomatic: interpreting meaning is central to translation (Noblit and Hare 1988). Reciprocal translation is used when primary studies are roughly about similar things (Noblit and Hare 1988, Britten and Pope 2012). The purpose of refutational translation is to explain and explore differences, incongruities and inconsistencies (Barnett-Page and Thomas 2009, Booth et al. 2013).  The various methods of conducting reciprocal translation have not been formally compared in methodological research. Common to the different reciprocal translation methods is a process of comparing the meaning of each concept (or theme or metaphor) from the primary studies to all the concepts from other studies in turn in order to arrive at new and/or combined overarching concepts (Atkins et al. 2008, Campbell et al. 2003, Campbell et al. 2006, Garside 2008, Pope and Mays 2006).  The eMERGe project found few published examples of refutational translation (Garside 2008, Wikberg and Bondas 2010). |
| 14 | Outcome of translation | Common pitfalls in published meta-ethnographies are: reviewers not clearly stating whose interpretation is being analysed or reported (France et al. 2014); and a lack of transparency in the development of a new interpretation/configuration of data (Kinn et al. 2013). There should be a “a clear auditable process linking findings to their originating studies…to assess the extent to which individual studies contribute to the synthesis, whether themes are present in multiple studies, particular findings are contradictory, or particular studies are outliers” (Booth et al. 2013, p.133). Reviewers should ensure that whose interpretation is being presented - that of the original research participants (sometimes called ‘first order constructs’), the authors of primary study accounts (‘second order constructs’), or the reviewers (‘third order constructs’) - is made clear for readers. |

| **Phase 6 – Synthesising translations** | | |
| --- | --- | --- |
| 15 | Synthesis process | Synthesising translations refers to “making a whole into something more than the parts alone imply… when the number of studies is large and the resultant translations numerous, the various translations can be compared with one another to determine if there are types of translations or if some metaphors and/or concepts are able to encompass those of other accounts” (Noblit and Hare 1988, p.29).  If few translated concepts arise (from phase 5) then it may not be possible to conduct a synthesis.  There is no single way to carry out the synthesis process – possible models include those by Atkins *et al* (2008), Britten *et al* (2002), Campbell *et al* (2011) and Toye *et al* (2014). How the synthesis of translations is conducted depends largely on the way translation was conducted. Translation and synthesis tend to happen simultaneously and in an iterative manner (Doyle 2003).  Line of argument can be described as a synthesis which links translations and the reviewers’ interpretation. Some clear and detailed examples of how line of argument synthesis has been conducted can be found in Britten *et al* (2002), Campbell *et al* (2003) and Malpass *et al* (2009).  The analysis and synthesis process appears to be best done collaboratively by a team (Atkins et al. 2008, Bondas and Hall 2007, Garside 2008, Toye et al. 2014) so that review findings are considered from alternative perspectives. |
| 16 | Outcome of synthesis process | The intention of meta-ethnography is to produce a new theory, interpretation or model, even if this was not ultimately possible (Atkins et al. 2008, Campbell et al. 2011, Malpass et al. 2009). Reviewers must be careful in stating that they are reporting new findings and be aware of the possible influence of findings from other authors on their own conclusions (Booth 2013). Sometimes a new interpretation might not be possible, for example, if ‘no new conceptual development had taken place following early conceptually-rich primary studies’ (France et al. 2014, p.11). |

**References**

Atkins, S., Lewin, S., Smith, H., Engel, M., Fretheim, A. & Volmink, J. (2008) Conducting a meta-ethnography of qualitative literature: lessons learnt. *BMC Med Res Methodol,* **8**, 21.

Barnett-Page, E. & Thomas, J. (2009) Methods for the synthesis of qualitative research: a critical review. *BMC Med Res Methodol,* **9**, 59.

Bondas, T. & Hall, E.O. (2007) Challenges in approaching metasynthesis research. *Qual Health Res,* **17**(1), 113-21.

Booth, A. (2013) Acknowledging a Dual Heritage for Qualitative Evidence Synthesis: Harnessing the Qualitative Research and Systematic Review Research Traditions Vol. PhD University of Sheffield. .

Booth, A., Carroll, C., Ilott, I., Low, L.L. & Cooper, K. (2013) Desperately seeking dissonance: identifying the disconfirming case in qualitative evidence synthesis. *Qual Health Res,* **23**(1), 126-41.

Britten, N., Campbell, R., Pope, C., Donovan, J., Morgan, M. & Pill, R. (2002) Using meta ethnography to synthesise qualitative research: a worked example. *Journal of Health Services & Research Policy,* **7**(4), 209-215.

Britten, N. & Pope, C. (2012) Medicine taking for asthma: a worked example of meta-ethnography (Chapter 3). (Hannes, K. and Lockwood, C. eds.) Wiley-Blackwell BMJ Books, Chichester, pp. 41-58.

Campbell, R., Britten, N., Pound, P., Donovan, J., Morgan, M., Pill, R. & Pope, C. (2006) Moving beyond effectiveness in evidence synthesis: Methodological issues in the synthesis of diverse sources of evidence. In *Section 4- Using meta-ethnography to synthesise qualitative research.*(Popay, J. ed. NICE, London, pp. 110.

Campbell, R., Pound, P., Morgan, M., Daker-White, G., Britten, N., Pill, R., Yardley, L., Pope, C. & Donovan, J. (2011) Evaluating meta-ethnography: systematic analysis and synthesis of qualitative research. *Health Technol Assess,* **15**(43), 1-164.

Campbell, R., Pound, P., Pope, C., Britten, N., Pill, R., Morgan, M. & Donovan, J. (2003) Evaluating meta-ethnography: a synthesis of qualitative research on lay experiences of diabetes and diabetes care. *Soc Sci Med,* **56**(4), 671-84.

Doyle, L.H. (2003) Synthesis through meta-ethnography: paradoxes, enhancements, and possibilities. *Qualitative Research,* **3**(3), 321-344.

Erasmus, E. (2014) The use of street-level bureaucracy theory in health policy analysis in low- and middle-income countries: a meta-ethnographic synthesis. *Health Policy Plan,* **29 Suppl 3**, iii70-8.

Finfgeld-Connett, D. (2014) Metasynthesis findings: potential versus reality. *Qual Health Res,* **24**(11), 1581-91.

France, E.F., Ring, N., Thomas, R., Noyes, J., Maxwell, M. & Jepson, R. (2014) A methodological systematic review of what's wrong with meta-ethnography reporting. *BMC Med Res Methodol,* **14**, 119.

Garside, R. (2008) A Comparison of methods for the Systematic Review of Qualitative Research : Two Examples Using Meta-Ethnography and Meta-Study. Vol. PhD University of Exeter.

Kangasniemi, M., Lansimies-Antikainen, H., Halkoaho, A. & Pietila, A.M. (2012) Examination of the phases of metasynthesis: a study on patients' duties as an example. *Prof Inferm,* **65**(1), 55-60.

Kinn, L.G., Holgersen, H., Ekeland, T.J. & Davidson, L. (2013) Metasynthesis and bricolage: an artistic exercise of creating a collage of meaning. *Qual Health Res,* **23**(9), 1285-92.

Lee, R.P., Hart, R.I., Watson, R.M. & Rapley, T. (2015) Qualitative synthesis in practice: Some pragmatics of meta-ethnography. *Qualitative Research,* **15**(3), 334-350.

Malpass, A., Shaw, A., Sharp, D., Walter, F., Feder, G., Ridd, M. & Kessler, D. (2009) "Medication career" or "moral career"? The two sides of managing antidepressants: a meta-ethnography of patients' experience of antidepressants. *Soc Sci Med,* **68**(1), 154-68.

Noblit, G.W. & Hare, R.D. (1988) *Meta-Ethnography: Synthesizing Qualitative Studies,* Sage Publications, California.

Pope, C. & Mays, N. (2006) Synthesising qualitative research. In *Qualitative research in health care (3rd ed.).*(Pope, C. and Mays, N. eds.) Blackwell Publishing;BMJ Books, Oxford UK, pp. 142-152.

Thorne, S., Jensen, L., Kearney, M.H., Noblit, G. & Sandelowski, M. (2004) Qualitative metasynthesis: reflections on methodological orientation and ideological agenda. *Qual Health Res,* **14**(10), 1342-65.

Toye, F., Seers, K., Allcock, N., Briggs, M., Carr, E. & Barker, K. (2014) Meta-ethnography 25 years on: challenges and insights for synthesising a large number of qualitative studies. *BMC Med Res Methodol,* **14**, 80.

Wikberg, A. & Bondas, T. (2010) A patient perspective in research on intercultural caring in maternity care: A meta-ethnography. *Int J Qual Stud Health Well-being,* **5**.
