# Supplementary material for: Improving reporting of meta-ethnography: the eMERGe reporting guidance
Source: BMC Med Res Methodol. 2019 Jan 31;19:25. doi: 10.1186/s12874-018-0600-0 (PMC6359764; doi:10.1186/s12874-018-0600-0)
Supplement: Supplementary file 5 — Part 3: eMERGe Reporting Guidance - Extensions. (DOCX 48 kb) [file 12874_2018_600_MOESM5_ESM.docx]

**Part 3: eMERGe Reporting Guidance - Extensions**

**Format and content of meta-ethnography outputs**

Published meta-ethnographies are often difficult to identify. One reason for this is that the term ‘meta-ethnography’ often does not appear in the titles or abstracts of journal papers or reports. In addition, our audit of published meta-ethnographies found that abstracts are often poorly reported and lack clarity for readers regarding their methods and findings.

Therefore, the following criteria should be considered:

- Include the term meta-ethnography in the title, abstract and/or keywords.

Reporting a meta-ethnography may take a number of formats including, for example, a journal paper, research report, policy document or film depending on the intended audience.

The abstract, lay summary and/or executive summary should be tailored to the intended audience. Ideally it should contain brief details of:

- the study’s background; aim and review question or objectives; search strategy; methods of selection, appraisal, analysis and synthesis of primary study accounts
- main findings including a description of the model, conceptual framework, or theory and the number of studies synthesised
- implications for policy, practice and/or theory.

Journal editors should note that reviewers might use a particular format, such as use of italics or alignment of text, to illustrate how the primary studies are related and it is important this is replicated exactly during the editing process as this affects interpretation of the material.

**Assessment of the methodological strengths and limitations of included primary studies**

Noblit and Hare (1988) did not consider the assessment of the methodological strengths and limitations of included primary studies in their original text. Whether to explicitly make such assessments remains a contentious issue amongst qualitative researchers partly because what is a strength in one type of qualitative research may be a limitation in another (Carroll and Booth 2015, Toye et al. 2014). Although many meta-ethnographies are commonly published without a formal appraisal of the methodological strengths and limitations of included studies (France et al. 2014), Campbell and colleagues identified important benefits when formal appraisal criteria were applied (Campbell et al. 2011). The appraisal process facilitated closer reading of studies to identify their methodological strengths and limitations, and aided interpretation of their potential contribution to the synthesis. Campbell concluded that ‘although there is an argument that including weak studies gives them an unwarranted credibility, such studies do not unduly distort a qualitative synthesis in the way that a poor-quality, highly biased quantitative study could influence a meta-analysis. In a qualitative synthesis, it is the power of ideas that matters.’ (Campbell et al. 2011, p.122).

If the findings of a meta-ethnography will be used in a decision-making context (such as an evidence-to-recommendation process undertaken by a clinical guideline development group) then an assessment of the methodological strengths and limitations of included studies is needed as part of an assessment of how much confidence can be placed in these findings (see GRADE CERQual extension below (Lewin et al. 2015)).

The Cochrane Qualitative and Implementation Methods Group provide detailed guidance on the selection and use of an appraisal tool for assessing methodological strengths and limitations (Noyes et al. 2018). The guidance covers key aspects of reviewer decision-making such as how and when to use the appraisal process to make inclusion, exclusion and sampling decisions relevant to the review question. In a meta-ethnography, appraisal of the methodological strengths and limitations of primary studies may also be carried out to identify conceptually rich papers. Some meta-ethnographies include relevant studies irrespective of their methodological limitations so reviewers need to consider how to use study assessments when determining and interpreting findings.

**Reporting of the assessment process should be transparent and document the rationale for decisions made. The following aspects should be considered:**

- provide a rationale for conducting / not conducting an assessment of methodological strengths and limitations.
- identify the assessment tool.

for each primary qualitative study, report in a table the assessment made for each domain of the tool used to assess methodological strengths and limitations. Consider including evidence (such as providing a succinct summary of each review finding) for these judgements.

- describe how the assessments were used in the meta-ethnography. For example: as a means of selecting primary studies, or as information to use when interpreting the findings etc.

**Using GRADE-CERQual to assess confidence in findings from qualitative evidence syntheses**

If the findings of a meta-ethnography will be used to inform health care decision-making then an assessment of confidence in the synthesised qualitative findings is important (Lewin et al. 2015). The GRADE-CERQual (Confidence in the Evidence from Reviews of Qualitative research) approach includes four components: the methodological limitations of the individual qualitative studies contributing to a review finding; the relevance to the review question of the individual studies contributing to a review finding; the coherence of the review finding; and the adequacy of data supporting a review finding. Detailed guidance on the application of CERQual is available elsewhere (Lewin et al. 2015), CERQual website: [www.cerqual.org](http://www.cerqual.org)).

There are however some important considerations when undertaking and reporting a meta-ethnography in a decision-making context. Meta-ethnography may produce two different levels of findings: in phases 3 to 5, metaphors, themes and concepts from across the included studies may be identified and synthesised in a reciprocal translation and refutational analysis. In phase 6 these themes and concepts are translated into one another to inform the development of broader concepts or theory. These different levels of findings may be useful at different stages of a decision-making process. For example, the broad concepts or theory emerging from a meta-ethnography may help shape a decision-making process by providing an explanation of a phenomenon or process, as experienced by stakeholders. The synthesised themes and concepts from the earlier phases of a meta-ethnography may inform specific decisions, such as whether an intervention is acceptable to stakeholders. In principle, CERQual can be applied to both levels of findings. The use of CERQual to assess more descriptive findings is now well established and guidance is available (Lewin et al. 2015). However, there is much less experience in applying CERQual to the broader concepts or theory that may emerge from a meta-ethnography and guidance on this is still to be developed.

**When reporting the application of CERQual in a meta-ethnography, the following considerations are important:**

- describe the meta-ethnography review question in detail, including the phenomena of interest and the relevant aspects of context (Noyes et al. 2018).
- undertake and report an assessment of the methodological limitations of the primary studies contributing to each finding, along with the assessments for the other three CERQual components.
- report the synthesised findings from primary studies, along with their CERQual assessments, in a Summary of Qualitative Findings Table. Examples can be found in Lewin et al (2015) and at the CERQual website.

**References**

Campbell, R., Pound, P., Morgan, M., Daker-White, G., Britten, N., Pill, R., Yardley, L., Pope, C. & Donovan, J. (2011) Evaluating meta-ethnography: systematic analysis and synthesis of qualitative research. *Health Technol Assess,* **15**(43), 1-164.

Carroll, C. & Booth, A. (2015) Quality assessment of qualitative evidence for systematic review and synthesis: Is it meaningful, and if so, how should it be performed? *Res Synth Methods,* **6**(2), 149-54.

France, E.F., Ring, N., Thomas, R., Noyes, J., Maxwell, M. & Jepson, R. (2014) A methodological systematic review of what's wrong with meta-ethnography reporting. *BMC Med Res Methodol,* **14**, 119.

Lewin, S., Glenton, C., Munthe-Kaas, H., Carlsen, B., Colvin, C.J., Gulmezoglu, M., Noyes, J., Booth, A., Garside, R. & Rashidian, A. (2015) Using qualitative evidence in decision making for health and social interventions: an approach to assess confidence in findings from qualitative evidence syntheses (GRADE-CERQual). *PLoS Med,* **12**(10), e1001895.

Noblit, G.W. & Hare, R.D. (1988) *Meta-Ethnography: Synthesizing Qualitative Studies,* Sage Publications, California.

Noyes, J., Booth, A., Flemming, K., Garside, R., Harden, A., Lewin, S., Pantoja, T., Hannes, K., Cargo, M. & Thomas, J. (2018) Cochrane Qualitative and Implementation Methods Group guidance series-paper 3: methods for assessing methodological limitations, data extraction and synthesis, and confidence in synthesized qualitative findings. *Journal of clinical epidemiology,* **97**, 49-58.

Toye, F., Seers, K., Allcock, N., Briggs, M., Carr, E. & Barker, K. (2014) Meta-ethnography 25 years on: challenges and insights for synthesising a large number of qualitative studies. *BMC Med Res Methodol,* **14**, 80.
